# Controllable surface carrier type of metal oxide nanocrystals for multifunctional photocatalysis

**DOI:** 10.1016/j.isci.2025.111750

**Published:** 2025-01-04

**Authors:** Han Li, Yingchun Ding, Kaiyi Luo, Qiuping Zhang, Huan Yuan, Shuyan Xu, Ming Xu

**Affiliations:** 1 College of Electronic Information & Key Lab of Information Materials of Sichuan Province, Southwest University for Nationalities, Chengdu 610041, China; 2 Department of Material and Chemical Engineering, Yibin University, Yibin 644000, China; 3 National Laboratory of Solid State Microstructures, Nanjing University, Nanjing 210093, China; 4 Plasma Sources and Application Center, Nanyang Technological University, 50 Nanyang Avenue, Singapore 637616, Singapore

**Keywords:** Catalysis, Engineering, Materials science

## Abstract

Selectively harnessing photo-induced carriers to control surface photo-redox reactions can enable currently limited specificity in photocatalytic applications. By using a new approach to switching between dominant electron and hole charge transfer on the surfaces of metal oxide nanocrystals, depending on the optimal carrier for specific application functionality in photocatalytic pollutant degradation, H_2_ production, CO_2_ reduction, and gas sensing. The approach is based on the surface redox properties of custom-designed p-n hetero-structured hybrid nanoparticles (NPs) containing copper oxide, and wide-gap metal oxide semiconductors (MOSs). The customized Cu_x_O/ZnO (CXZ) heterostructures ensure effective charge separation and surface reactions driven by UV-vis excited highly reactive holes and show high performance in the photo-oxidative degradation of organic dyes and NO_2_ gas sensing. By switching the dominant surface carrier type from holes to electrons, the hybrids exhibit excellent performance in photocatalytic H_2_ evolution and CO_2_ reduction. This work offers a generic approach to engineering multipurpose photocatalytic materials.

## Highlights

• p-n hetero-structures containing copper oxide and wide-gap metal oxide were prepared
• Switching between dominant electron and hole on the metal oxide surface is controllable
• Synthesized heterostructures favor surface oxidation reactions driven by holes
• Re-engineered nanostructures exhibit strong photocatalytic reduction by electrons

## Graphical abstract

**Figure:**
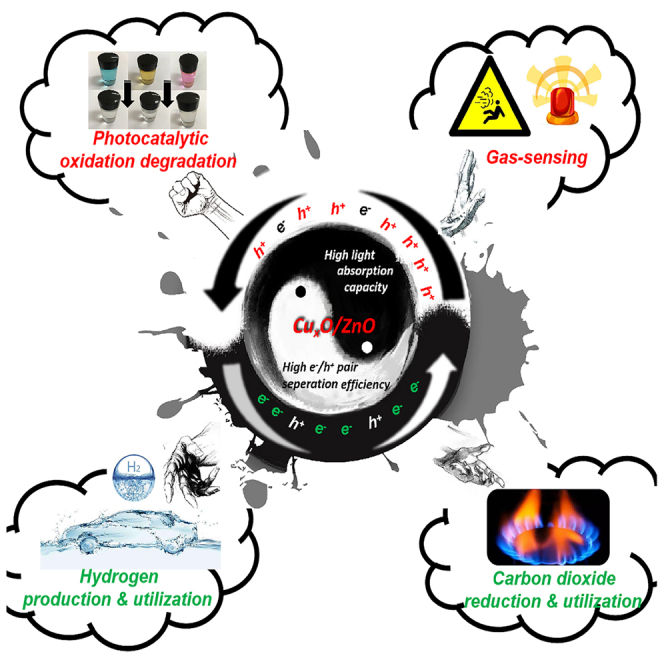

## Introduction

Economic development significantly relies on natural resources, and society is increasingly affected by the environmental and energy crisis.^1^^,^^2^ Photocatalytic technologies may contribute to resolving environmental and clean energy challenges.^3^^,^^4^ Conventional wide-bandgap metal oxide semiconductors (MOSs), such as zinc oxide (ZnO), titanium dioxide (TiO_2_), tin dioxide (SnO_2_), and zirconium dioxide (ZrO_2_) have been extensively used due to their high activity and stability.^5^^,^^6^^,^^7^^,^^8^ However, these materials cannot overcome the issues of limited visible light absorption capacity and short photo-generated charge lifetime when used as a single catalytic platform. Therefore, the development of MOS photocatalysts with high light-harvesting and charge separation efficiency has recently attracted significant interest.

Co-catalysts play several critical roles in photocatalysis. Noble metal co-catalysts such as Pd, Pt, Au, and Ag on semiconductor supports promote photocatalytic H_2_ evolution as well as the conversion of CO_2_ and H_2_O to hydrocarbons, and ensures the high-efficiency removal of organic dyes and hazardous gases.^8^^,^^9^ However, the high cost of noble metals greatly limits their practical application.

Active species, such as **·**O_2_^−^, H_2_O_2_, ^1^O_2_, **·**OH, and photogenerated holes (h^+^) play an important role in photocatalytic oxidation, with h^+^ and **·**OH radicals being particularly energetic. In contrast, photocatalytic reduction is driven by electrons (e^−^) which migrate to the surface. Certain *p-n* type materials can simultaneously photo-oxidize organic contaminants and photo-reduce metallic ions due to their enhanced visible light absorption and charge transfer efficiency.^10^^,^^11^ However, the ability to effectively control the dominant type and transport of photo-generated charge carriers has remained unexplored. This is critical to obtaining the currently elusive ability to switch between dominant photocatalytic oxidation or reduction reactions on the catalytic surface, similar to the highly selective operation of transistors in microelectronics. To the best of our knowledge, no reports have been published on obtaining high catalytic efficiency in a broad range of photocatalytic processes by switching the dominant type of photo-excited carriers on the surface of the same nanoparticle (NP) material. Therefore, we demonstrated this possibility for the first time through simple adjustments to the preparation processes of CuxO/MOS heterostructures and provided new insights into the mechanisms of the resulting photocatalytic effects.

**Figure 1:**
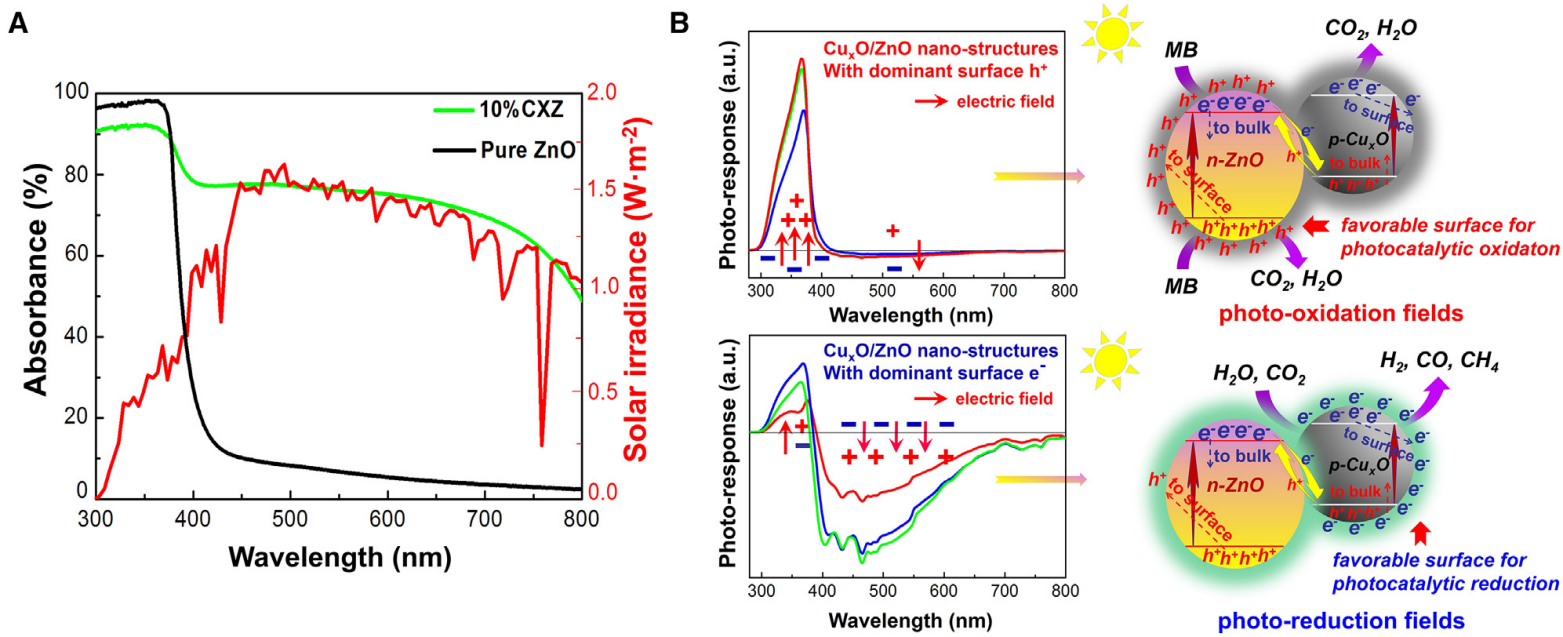
Optical properties and dominant surface carrier types of Cu_x_O/MOS heterostructures (A) Cu_x_O NPs could act as a co-catalyst and a visible-light-harvesting medium. (B) Switching the dominant surface carrier types could produce high performance in multiple photocatalytic applications.

Our solution involved copper oxide (Cu_x_O, CuO, E_g_ = 1.7 eV; Cu_2_O, E_g_ = 2.0 eV) co-catalysts that featured visible-light-active properties and high performance in photocatalytic oxidation and reduction.^12^^,^^13^ A series of Cu_x_O/MOS heterostructures, including Cu_x_O/ZnO, Cu_x_O/TiO_2_, Cu_x_O/SnO_2_, and Cu_x_O/ZrO_2_ with significantly enhanced light-harvesting capacity were synthesized using simple methods (Figure 1A). We revealed that the surface photo-excited charge carriers were crucial for inducing the photocatalytic preference of heterogeneous nanoparticle materials. Specific means to regulate the major surface photo-generated carrier types were subsequently demonstrated, enabling the catalysts to steer the targeted oxidation/reduction reactions and improve photocatalytic performance. The sol-gel-based Cu_x_O/MOS heterostructures, especially the Cu_x_O/ZnO (CXZ) NPs, demonstrated high photo-activity in the degradation of organic dyes due to efficient photo-excited e^−^/h^+^ pair separation, as well as the directional migration of strongly oxidizing h^+^ to the catalyst surface. Subsequently, the major surface carrier type was changed by fabricating the heterostructures employing a polymer network gel route, with the heterostructures being electronically dominated on the surface under visible light excitation. The remarkable surface photo-reduction effect enabled the re-engineered NPs to undergo efficient photocatalytic H_2_ production and CO_2_ reduction. This distinction is mainly originated from the diversity in the donor impurity concentration brought about by the different annealing temperatures of the two methods. The annealing temperature of 450°C for the sol-gel method results in the high concentration of donor impurity in the matrix crystals, leading to the stronger surface n-type conductivity and higher electric field neutralization effect. In contrast, the CuxO/MOSs(r) samples obtained at higher annealing temperatures (650°C) with a lower donor defect concentration possess stronger p-type conductivity and a weaker electric field offset effect, allowing a considerable number of electrons to migrate to the surface. Therefore, this work offers a versatile solution for the development of cost-effective visible-light-active photocatalysts that can selectively drive diverse reactions for various practical applications (Figure 1B).

## Results

### Characterization of sol-gel-based Cu_x_O/MOS nanoparticle photocatalysts

Figure S1A shows the X-ray diffraction (XRD) spectrum of the as-fabricated Cu_x_O. The diffraction peaks marked by “∗” located at 32.65°, 35.58°, 38.84°, 48.95°, 53.67°, 58.24°, 61.66°, 66.39°, and 68.18° corresponded to the (−110), (002), (111), (20–2), (112), (020), (202), (11-3), (310), and (220) crystal planes of tenorite CuO (JCPDF #48-1,548), respectively. In addition, the peaks denoted by “&,” which were located at 29.79°, 36.60°, and 42.48°, were assigned to the (100), (111), and (200) crystal planes of Cu_2_O, respectively (JCPDF #34-1,354). XRD analysis indicated that Cu^+^ (Cu_2_O) and Cu^2+^ (CuO) co-existed in the copper oxides. The morphology of the Cu_x_O powder was characterized by scanning electron microscopy (SEM) and transmission electron microscope (TEM), indicating that the Cu_x_O NPs had a grain-like structure, as shown in Figures S1B and S1C. The UV-vis absorption spectrum (Figure S1D) demonstrated the high absorption capacity of Cu_x_O for UV and visible light, especially in the visible region (from ∼400 to ∼700 nm). Figure S1E shows the surface photo-voltage (SPV) response of Cu_x_O, indicating that the Cu_x_O NPs showed almost no SPV signals due to the rapid recombination of the photo-excited e^−^/h^+^ pairs. As displays in Figure S1F, the electron paramagnetic resonance (EPR) results indicate the presence of copper vacancies in Cu_x_O.^14^

**Figure 2:**
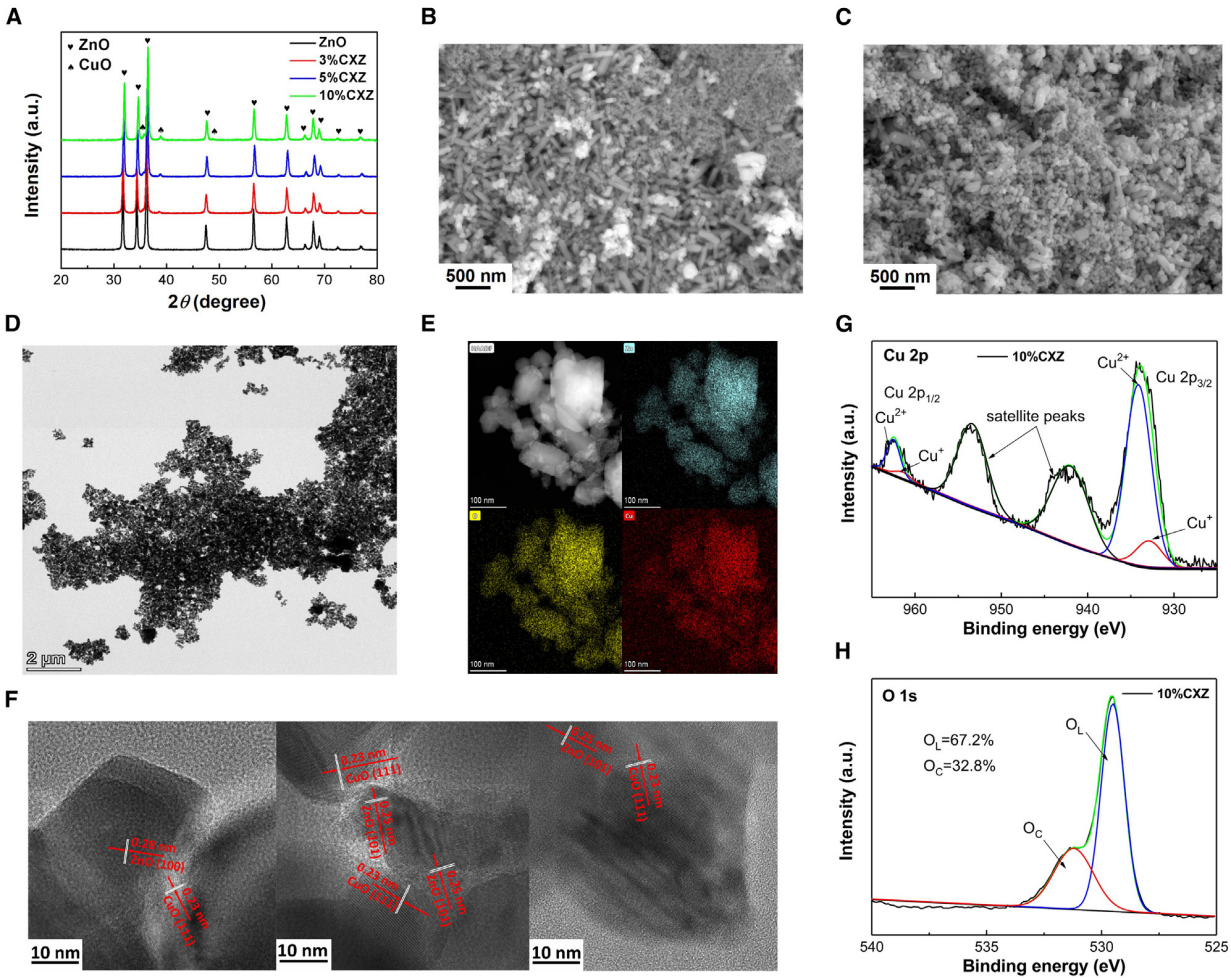
Microstructures and compositions characterization of Cu_x_O/ZnO (A) XRD patterns of pure ZnO and Cu_x_O/ZnO samples. (B and C) SEM images of 3%CXZ. (D) TEM image of 3%CXZ. (E) EDS elemental mapping image of 3%CXZ. (F) HRTEM images of 3%CXZ. (G and H) High-resolution XPS spectra of 10%CXZ (Cu 2p, O 1s).

XRD was used to analyze the phase structures of all the Cu_x_O/MOS samples prepared by the sol–gel method, as shown in Figures 2A and S2A–S2C. Taking Cu_x_O/ZnO as an example (Figure 2A), all samples demonstrated good crystallinity, and we identified the hexagonal wurtzite structure type of ZnO (JCPDF #65-3,411). The microstructure parameters calculated according to XRD for the Cu_x_O/ZnO samples are shown in Table S1, and the morphology and distribution of the Cu_x_O/ZnO nanocrystals were examined by SEM and TEM. Figures 2B and 2C presents the SEM images of the 3%CXZ sample, and we observed no apparent agglomeration of the ZnO NPs. Figures 2D and 2E shows the TEM images and elemental dispersion spectral (EDS) results for the 3%CXZ sample, which indicated the uniform grain size and elemental distribution. High-resolution transmission electron microscopy (HRTEM, Figure 2F) revealed the formation of Cu_x_O/ZnO heterojunctions. The interplanar lattice spacings of 0.28 and 0.25 nm perfectly corresponded to the (101) and (002) crystal planes of ZnO, and the interplanar lattice spacing of 0.23 nm corresponded well to the (111) crystal plane of CuO, indicating the formation of Cu_x_O/ZnO heterostructure. We used X-ray photoelectron spectroscopy (XPS) to investigate the chemical states of the photocatalyst. As shown in Figure 2G, the strong satellite peaks indicated the presence of Cu^2+^. Moreover, the presence of Cu^+^ can be ascertained by fitting the Cu 2p signal into Gaussian peaks. Figure 2H displays the proportion of the O atoms distributed in lattice (O_L_) and on the material surface (chemisorbed oxygen, [O_C_]), suggesting that the number of O_L_ atoms was ∼2.0 times higher than the O_C_ atoms.

**Figure 3:**
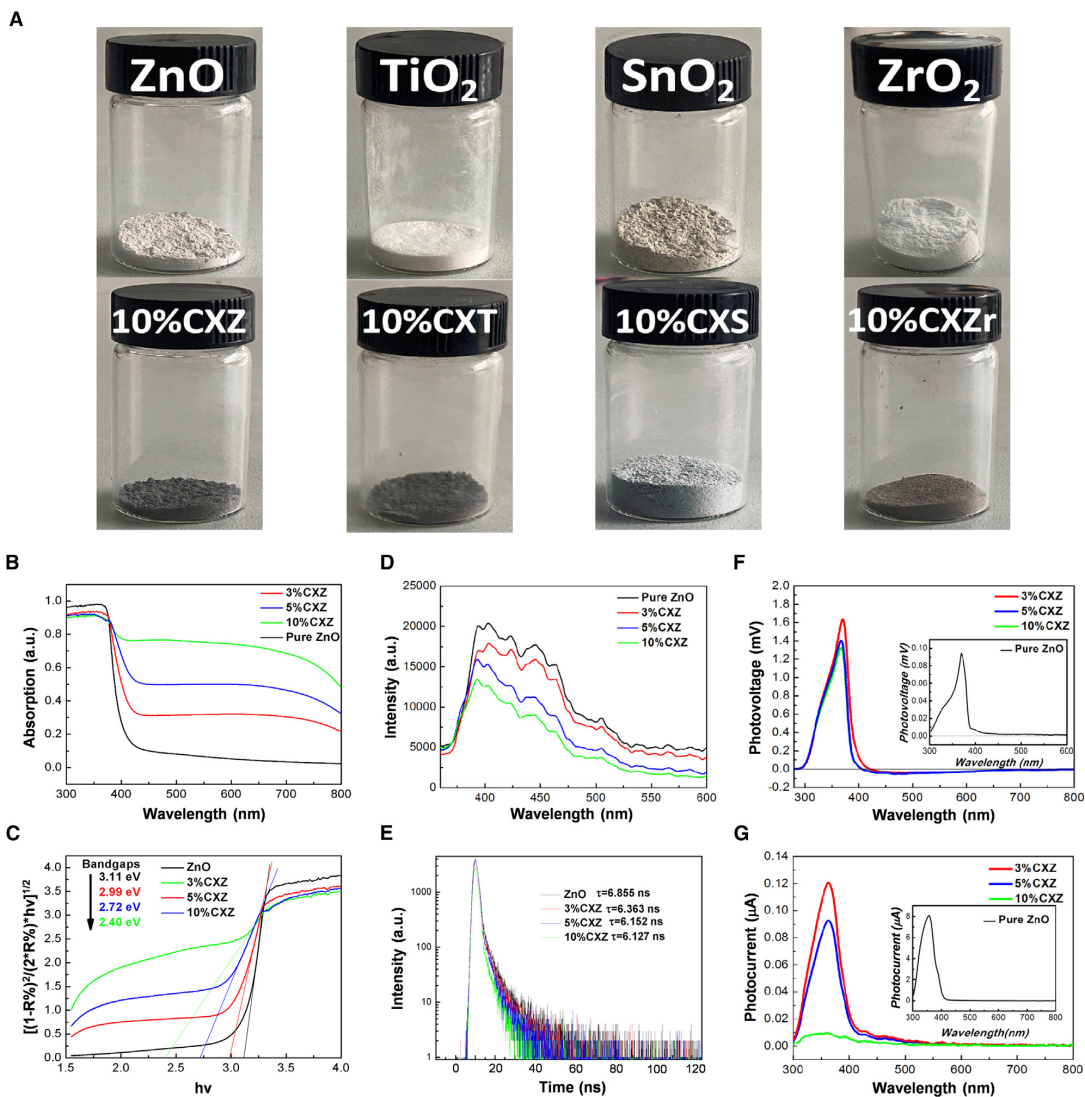
Photoelectric properties characterization of Cu_x_O/ZnO (A) Comparison of appearance before and after introducing Cu_x_O. (B) UV-vis absorption spectra. (C) Optical bandgaps of calculated by the Kubelka-Munk theory and the Tauc plot. (D) Steady-state PL spectra. (E) Time-resolved PL spectra. (F) Steady-state SPV spectra. (G) Steady-state SPC spectra.

The Cu_x_O/MOS samples changed in color from light to dark with the introduction of Cu_x_O (Figure 3A), implying that the samples coupled with Cu_x_O may possess a high absorption capacity to visible light. As shown in Figure 3B, the introduction of Cu_x_O NPs dramatically enhanced the solar absorption in the visible range from ∼400 to 800 nm, and narrowed the band gap width of pure ZnO to a large extent (Figure 3C), although there was a slight reduction in UV light absorption. Similar enhancements were also observed on the other Cu_x_O/MOS samples (Figures S2D–S2F).

To investigate the separation efficiency and lifetimes of the photo-excited carriers, steady-state photoluminescence (PL), and time-resolved PL measurements were employed. The steady-state PL spectra of the Cu_x_O/ZnO samples (Figure 3D) demonstrated significantly reduced emissions in both the UV and visible regions compared to pristine ZnO, implying the effective recombination suppression of the photo-excited e^−^/h^+^ pairs. Figure 3E shows the time-resolved PL curves for the Cu_x_O/ZnO samples, where the shorter fluorescence lifetime indicated that efficient charge transfer occurred in the Cu_x_O-decorated samples, which was consistent with recent reports.^15^^,^^16^^,^^17^ SPV measurements further demonstrated the prolonged lifetime of the photo-excited e^−^/h^+^ pairs. As shown in Figure 3F, the SPV response of Cu_x_O/ZnO at ∼370 nm prominently increased from +0.09 mV (ZnO) to +1.41 mV (3%CXZ). The as-prepared Cu_x_O exhibited no SPV signal (Figure S1E), however, for the Cu_x_O/ZnO heterostructures, weak negative SPV signals (reaching approx. ˗0.05 mV) were observed in the visible region. Theoretically, ZnO can serve as an n-type semiconductor with an upward surface band bending, and the built-in electric field could be directed from the bulk toward the surface in the surface space charge region, and thus, the photo-excited holes of ZnO could move to the surface and a positive photovoltaic signal was generated. However, CuO and Cu_2_O were p-type semiconductors due to the presence of copper vacancies, the built-in electric field was oriented from the surface toward the bulk.^18^ When Cu_x_O was excited, photo-excited electrons transferred to the surface and a negative photovoltage response occurred. The surface photo-current (SPC) responses (Figure 3G) of the Cu_x_O/ZnO nanostructures were significantly reduced compared to pure ZnO due to the formation of p-n junctions, which greatly inhibited free charge mobility.

### Photocatalytic oxidation properties for removing organics

The photocatalytic oxidation performance of the Cu_x_O/MOSs was evaluated by the photocatalytic degradation of methylene blue (MB) under simulated solar light irradiation, as shown in Figures S3A–S3D. The photocatalytic performance of the Cu_x_O/MOS samples was significantly enhanced compared to MOSs. The Cu_x_O/ZnO photocatalysts demonstrated superior performance for photo-oxidation degradation due to the high visible light absorption capacity and longer photo-induced charge lifetime. Specifically, the degradation rate of 3%CXZ for MB exceeded ∼93% in only 10 min, and the first-order kinetic degradation constant [*K*×t = ln(C_0_/C_t_)] was ∼0.3, which was approximately 6.4 times that of pure ZnO (0.047). Re-engineered Cu_x_O/MOS heterostructures (labeled as Cu_x_O/MOS(r)) were fabricated via the polymer network gel method. In the case of Cu_x_O/ZnO(r), for example, XPS characterization (Figure S4) showed no significant change in the valence state ratio of the surface Cu ions after re-engineering, and the increase in the ratio of lattice oxygen relative to surface adsorbed oxygen indicated an enhancement in crystallinity.^19^ The removal results of MB under simulated sunlight irradiation with Cu_x_O/ZnO(r) are shown in Figure S3E. The Cu_x_O/ZnO(r) samples showed only a slight improvement in catalytic activity over ZnO, though the 5%CXZ(r) sample demonstrated optimal performance, and the kinetic degradation constant was only ∼1.6 times higher than ZnO (K_5%CXZ(r)_ = 0.0760, K_ZnO(r)_ = 0.0465).

**Figure 4:**
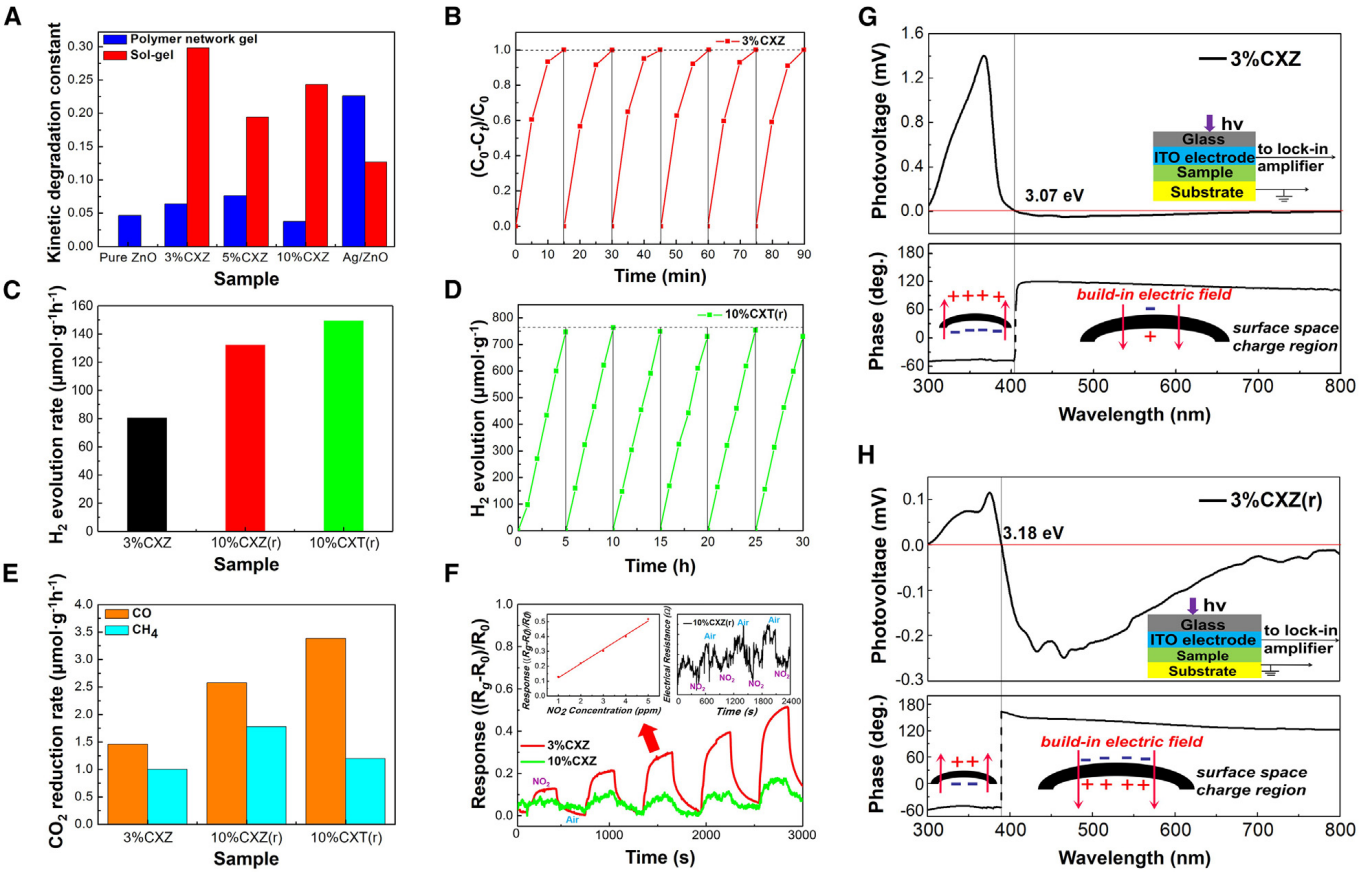
Control of the surface photo-responses and photocatalytic performance of pristine and re-engineered Cu_x_O/ZnO samples in diverse applications (A) Photocatalytic degradation of MB using Cu_x_O/ZnO, Cu_x_O/ZnO(r), and Ag/ZnO under simulated sunlight irradiation (dosage: 50 mg). (B) Cycling experiments of photocatalytic degradation of MB with 3%CXZ. (C) Photocatalytic H_2_ evolution on 3%CXZ, 10%CXZ(r), and 10%CXT(r) under simulated sunlight irradiation (dosage: 50 mg; irradiation time: 4 h). (D) Cycling experiments of photocatalytic H_2_ evolution on 10%CXT. (E) Photocatalytic CO_2_ reduction on 3%CXZ, 10%CXZ(r), and 10%CXT(r) under simulated sunlight irradiation (dosage: 50 mg; irradiation time: 4 h). (F) Real-time response curves of the sensors based on 3%CXZ, 10%CXZ(r), and 10%CXT(r) to NO_2_ gas with concentrations ranging from 1 to 5 ppm under 365-nm light irradiation at room temperature. (G and H) Representative SPV responses of Cu_x_O/ZnO (3%CXZ sample) and re-engineered Cu_x_O/ZnO (3%CXZ(r) sample), reflecting the distinct photo-response characteristics caused by the different dominant surface conductivity.

Furthermore, as shown in Figure S5, the 3 mol % Ag-decorated ZnO samples, which exhibited high photocatalytic degradation performance,^20^ were prepared and used to compare the effects of copper oxides and noble metal as co-catalysts. The results suggested that the degradation performance of 3%CXZ exceeded Ag/ZnO (K_Ag/ZnO_ = 0.1065, K_Ag/ZnO(r)_ = 0.1638), and this confirmed copper oxides as appropriate alternatives for noble metals. Figure 4A shows a comparison of MB degradation efficiency between Cu_x_O/ZnO and Cu_x_O/ZnO(r), suggesting that the 3%CXZ sample presented the highest photo-activity. A comparison of the XRD as well as XPS analyses and cyclic degradation properties, as shown in Figures S6 and 4B, shows an almost identical result before (fresh 3%CXZ, labeled as CXZ(f)) and after (used 3%CXZ, labeled as CXZ(u)) six degradation cycles, which demonstrate the excellent stability of the prepared nanocomposites. In addition, the high-resolution Cu 2p profiles in Figure S6C show that the valence states of Cu remained unchanged during the photocatalytic process.

### Photocatalytic reduction performance

As shown in Figures S7A and 4C, photocatalytic H_2_ evolution was measured under simulated sunlight excitation to investigate the light-induced photo-reduction activities of the samples. Considering the strong performance inequality between the photocatalytic oxidation activities of the 3%CXZ and 10%CXZ(r) samples, our experiments focused on the 3 mol % and 10 mol % Cu_x_O samples based on two methods to assess the differences in photocatalytic reduction. Although the 3%CXZ sample demonstrated very strong activity for removing organics, the H_2_ formation rate exceeding 3%CXZ was only 80.51 μmol g^−1^·h^−1^. By contrast, the re-engineered heterostructure 10%CXZ(r), which showed poor photocatalytic degradation performance, exhibited notable photocatalytic H_2_ production capacity due to prominent surface photo-reduction properties. The H_2_ formation rate over the 10%CXZ(r) sample was 132.30 μmol g^−1^·h^−1^, which was around 1.6 times higher than that of 3%CXZ. Moreover, a similar contrast was found for the 10%CXT(r) sample (149.41 μmol g^−1^·h^−1^), and the six testing cycles over the 10%CXT(r) sample suggested good stability during photocatalytic H_2_ production (Figure 4D).

An evaluation of the simulated sunlight-driven photocatalytic CO_2_ reduction properties of the samples was performed to further compare the surface photo-reduction capacity of the Cu_x_O/ZnO and Cu_x_O/ZnO(r) catalysts (Figures S7B and S7C). Similarly, the re-engineered samples exhibited higher CO_2_ reduction performance. As shown in Figure 4E, the values of CO and CH_4_ generated over the 10%CXZ(r) sample were 2.58 and 1.78 μmol g^−1^·h^−1^, which was ∼1.8 times higher than over the 3%CXZ sample. Interestingly, the selectivity for CO_2_ reduction reached a high value (∼74%) with the 10%CXT(r) sample (Y_CO_ = 3.37 μmol g^−1^·h^−1^, Y_CH4_ = 1.20 μmol g^−1^·h^−1^). Therefore, the Cu_x_O/TiO_2_(r) heterostructure was possibly more favorable for photocatalytic H_2_ evolution and CO_2_ reduction than Cu_x_O/ZnO(r).

### Gas-sensing performance evaluation

We also assessed the CXZ heterostructures as active materials for light-sensitive metal-oxide-based gas sensors, which have recently attracted significant attention. Figure 4F shows the sensitivities [S = (R_g_ – R_0_)/R_0_] of the sensors based on different samples for low-concentration NO_2_ species under 365 nm illumination at room temperature. When NO_2_ was injected, the gas response of the sensors based on the 3%CXZ and 10%CXZ samples increased, and then rapidly decreased to a baseline as NO_2_ was replaced by air. The responses became enhanced when the NO_2_ concentration increased from 1 to 5 ppm. Overall, the sensor based on the 3%CXZ sample demonstrated the best sensitivity due to its superior charge carrier separation efficiency. Subsequently, a linear relationship between the sensing response and NO_2_ concentration (left image in Figure 4F) as well as the cycling experiments of NO_2_ gas-sensing at 5 ppm for the 3%CXZ sample (Figure S8) were investigated, which indicated high stability. However, for the sensor based on the 10%CXZ(r) sample under UV light excitation, a weak negative response was detected when NO_2_ was injected (right image in Figure 4F). This observation was most likely due to the Cu_x_O-driven major surface p-type conductivity of the re-engineered Cu_x_O/ZnO(r) heterostructures.

## Discussion

Previous studies have suggested that p-n heterojunction interfacial nanocomposites integrating zinc oxide and narrow-bandgap copper oxide can improve solar energy harvesting and charge carrier lifetime, enhancing photocatalytic performance.^21^^,^^22^^,^^23^^,^^24^ In this work, heterostructured NPs with the same composition were found to exhibit different surface photocatalytic trends following simple structural re-engineering. The performance of these NPs after photocatalytic oxidation and reduction was competitive with existing state-of-the-art research. As shown in Table S2, the performance of Cu_x_O/MOS and Cu_x_O/MOS(r) reaches a high level in terms of photocatalytic organic degradation, photocatalytic H_2_ production, and CO_2_ reduction without noble metal introduction. These findings indicate that copper oxides could serve as superior co-catalysts, verifying that the specific functionality of the catalysts promoted their targeted and highly efficient applications in various fields. We subsequently explored the mechanisms and driving factors of the distinct photocatalytic behaviors.

Figures 4G and 4H show the representative SPV responses of the Cu_x_O/ZnO and Cu_x_O/ZnO(r) catalysts to UV-visible light for the 3%CXZ and 3%CXZ(r) samples, with the spectra of all samples shown in Figure S9 of the electronic supplementary information (ESI). The lower panels of the images demonstrate the phase value as a function of wavelength. As shown in Figure 4G, for the CXZ sample, the photovoltage phases are distributed in the fourth quadrant and strong positive signals are generated under UV light excitation, indicating that photo-excited holes accumulated at the irradiation side. Under visible light illumination, the photovoltage phases are distributed in the second quadrant and weak negative photovoltage signal forms, yielded by Cu_x_O, which indicate a small number of photo-generated electrons accumulated at the irradiation side. In stark contrast, the re-engineered sample demonstrates a strongly negative SPV visible-light response with the integral area exceeding the positive UV-light response of ZnO (Figure 4H). These results demonstrate that the Cu_x_O/ZnO(r) sample mainly shows p-type conductivity and the surface is dominated by e^−^ rather than h^+^ under UV-visible light illumination, with the catalysts possibly possessing high surface photo-reduction activity. In the SPV results for CuxO/MOS heterostructures, the concave–convex shape of the peaks near ∼450 nm indicates that the negative signals in this range are not solely due to band-to-band transitions. Considering that the Cu vacancies caused the appearance of acceptor levels, it is possible that the visible-light responses are also relevant to the surface-state population transitions related to the acceptor defects of CuO and Cu_2_O nanocrystals, specifically, photo-generated electrons transitioned from the valence bands (VBs) to the acceptor levels, or from the CBs to the acceptor levels, and the associated built-in electric field is oriented from the surface toward the bulk.^18^^,^^25^ In general, the SPV responses of the same materials produced by different methods originate from the disparate major n-type and p-type conductivities of the composite photocatalysts. The sol-gel-based catalysts have dominant n-type conductivity, while the re-engineered samples showed dominant p-type conductivity, as evidenced by the opposite response trends in the gas-sensing results (Figure 4F).

The active species capturing experiments were performed to elucidate the effects of different active species during MB degradation with 3%CXZ. Benzoquinone (BQ), isopropanol (IPA), and ethylenediaminetetraacetic acid disodium salt (EDTA-2Na) were used as quenchers of superoxide radicals (**·**O_2_^−^), hydroxyl radicals (**·**OH), and photogenerated h^+^, respectively. As shown in Figure S10A, the degradation efficiencies of 3%CXZ in 15 min decrease from 100% to 68.9%, 18.4%, and 34.8% with the addition of BQ, IPA, and EDTA-2Na, illustrating that **·**O_2_^−^, **·**OH, and photogenerated h^+^ all participate in the photocatalytic decomposition of MB by CXZ nanocomposite. Among them, **·**OH plays the most important role and the trapping of h^+^ resulted in a relative noticeable decrease in the decontamination pace. In order to further demonstrate the existence of **·**OH, the EPR analysis was performed. As displayed in Figure S10B, DMPO-**·**OH signals are successfully detected under simulated light irradiation, further proving that the generation of **·**OH specie in the photocatalytic oxidation.

According to the concept of electronegativity,^26^ the edge positions of VB and conduction band (CB) for ZnO were estimated to be ca. +2.83 eV and −0.27 eV in this work. Comparing the VBs of ZnO and Cu_x_O (CuO, +0.67 eV; Cu_2_O, +0.53 eV),^27^^,^^28^ only the oxidation potential of h^+^ in ZnO is sufficiently large to oxidize OH^−^ and H_2_O to **·**OH (the potentials of OH^−^/**·**OH and H_2_O/**·**OH are +1.99 and +2.27 eV vs. normal hydrogen electrode (NHE), respectively).^29^ In addition, the h^+^ can directly oxidize adsorbate pollutants as a strong oxidant. Due to the low CB potential of ZnO, the e^−^ remained cannot effectively reduce oxygen molecules to **·**O_2_^−^ according to the potential of O_2_/**·**O_2_^−^ (−0.33 V vs. NHE). Compared to the response of ZnO to UV light, Cu_x_O shows a relatively weak response to visible light. Hence, the production of **·**O_2_^−^ in the photocatalytic oxidation process is mainly attributed to the e^−^ gathered in the CBs of Cu_x_O. Ultraviolet photoelectron spectroscopy (UPS) was performed to determine the work functions of the ZnO and Cu_x_O to understand the electron transfer behavior. As shown in the Figure S11, the energy of the secondary electron cut-off edge of ZnO, ZnO(r), and Cu_x_O are 17.56, 17.51, and 16.58 eV. The ultraviolet excitation energy is 21.22 eV, therefore the work functions are 3.66, 3.71, and 4.64 eV, respectively. The lower the work function of a material, the easier it is to donate electrons when in contact with other materials.^30^ Therefore, e^−^ will flow from ZnO or ZnO (r) to Cu_x_O under light excitation, and h^+^ will flow in opposite direction, effectively suppressing the recombination of photogenerated e^−^/h^+^ pairs. The results indicate that the photo-oxidation activity of the Cu_x_O/ZnO heterostructures mainly develops from photo-excited oxidizing h^+^ with longer lifetimes in ZnO.

**Figure 5:**
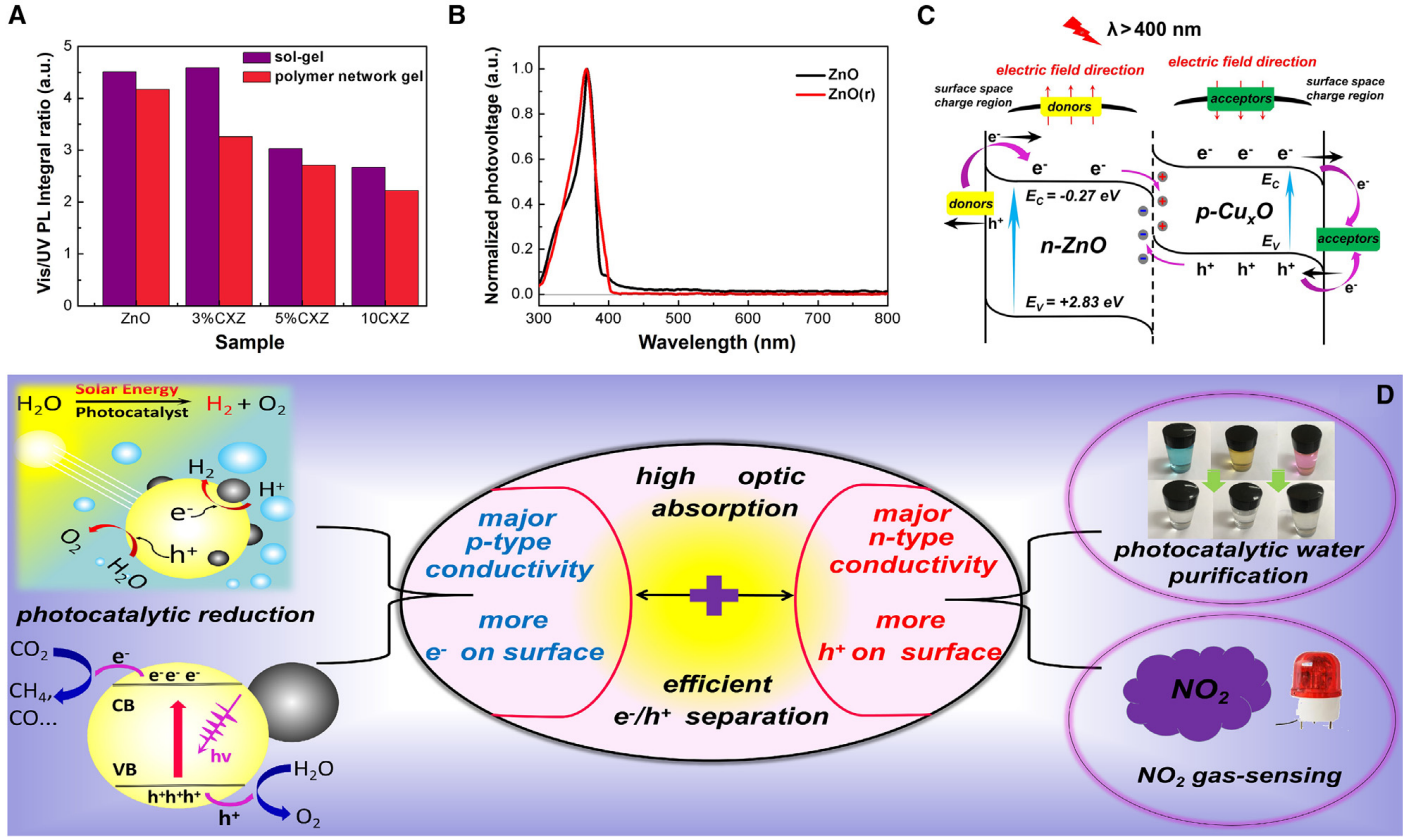
Mechanisms of selective photo-responses to visible light (A) Ratios of the integral area of visible-light emission to UV emission (analyzed from the PL results) for the two samples prepared by different methods. (B) Normalized photovoltages of ZnO and ZnO(r). (C) Schematic showing electrons flow on the surfaces of the composite materials under visible light excitation. (D) Multiple photocatalytic applications of the custom-engineered nanoparticle hetero-structured materials.

The evaluation of crystal quality and defect concentration in the materials could help us better understand the origin of strong and weak responses driven by visible light. The photocatalysts based on sol-gel method and polymer network gel method were annealed at 450°C and 650°C, respectively, and Figure S12 shows a comparison of the PL spectra of the Cu_x_O/ZnO and Cu_x_O/ZnO(r) samples. As shown in Figure 5A, the ratio of visible light emission integral area to UV-light emission integral area of Cu_x_O/ZnO is clearly larger than that of Cu_x_O/ZnO(r). This difference is mainly due to the crystalline quality improvement resulting from the higher annealing temperature, which also reduces donor impurity (i.e., Zn_i_, V_Zn_) concentration in the ZnO crystals. Because the ZnO crystallized during the second step of the experimental process, it is reasonable to assume that disparity occurred on the ZnO nanocrystals is obtained from different annealing temperatures. Hence, we fabricated pure ZnO again using the polymer network gel method under an annealing temperature of 650°C, with the samples labeled as ZnO(r). As shown in Figure 5B, the visible-light SPV responses of the pure ZnO and ZnO(r) are clearly different. The sol-gel-based ZnO presents a clear visible-light response, which likely originates from the sub-bandgap transitions of ZnO (depopulation transition of donor energy levels such as Zn_i_ and V_Zn_) and the built-in electric field is therefore oriented from the bulk toward the surface. As a result, the internal electric fields that aligned in opposite directions could offset each other within the composites. When the incident energy reaches a critical value, the built-in electric fields in the opposite directions with the same absolute value are completely canceled out, and the SPV response is equal to zero. Therefore, in the Cu_x_O/ZnO samples with a higher donor impurity concentration, which exhibits stronger surface n-type conductivity and high electric field neutralization effect, only a small fraction of electrons could migrate to the surface under visible light irradiation. However, the Cu_x_O/ZnO(r) samples with a lower donor defect concentration possess the stronger p-type conductivity and a weaker electric field offset effect. In this case, a considerable number of electrons could migrate to the surface. Based on the band gaps of ZnO, CuO, and Cu_2_O as well as their levels vs. NHE, a schematic of the plausible electron flow on the material surface under visible light illumination (λ > 400 nm) is obtained, as displayed in Figure 5C.

The rational introduction of defects into the ZnO nanocrystals to modulate the electronic properties and charge transport could enhance the photocatalytic degradation of pollutants or gas-sensitive response.^31^^,^^32^^,^^33^ Theoretical calculations were further used to reveal the effect of defects on the water decomposition and CO_2_ reduction reaction at the Cu_x_O/ZnO surface. Figure S13A shows the Gibbs free energy maps of the photocatalytic water splitting (left) and CO_2_ reduction (right) processes simulated on the surfaces of the defect-free Cu_x_O/ZnO materials and defect-rich nanocomposites (Cu_x_O/ZnO@De). The Gibbs free energy is a fundamental thermodynamic parameter that quantifies the likelihood that chemical reactions will occur. In the context of photocatalysis, the Gibbs free energy of key reactions, such as water splitting and carbon dioxide reduction, could provide valuable insights into the energy landscape of the catalytic process. The defect-rich Cu_x_O/ZnO nanocomposites exhibit a distinct advantage in terms of Gibbs free energy when compared to the defect-free counterparts. This advantage comes from the modified surface chemistry and electronic structure induced by the intentionally formed defects. Specifically, defects created localized states within the material’s bandgap, which could serve as intermediate energy levels for electron transfer during the catalytic reactions. In water splitting, for example, the formation of hydrogen and oxygen involved multistep proton-coupled electron transfer reactions. Defects in the nanocomposites facilitate these reactions by providing additional energy states that lowers the activation energy barriers. As a result, the overall Gibbs free energy change for water splitting becomes more favorable in the Cu_x_O/ZnO@De nanocomposites. Similarly, in carbon dioxide reduction, defects act as catalytic centers for the conversion of carbon dioxide into valuable hydrocarbons. The presence of defects enhances the adsorption of carbon dioxide molecules onto the catalyst surface and promoted the subsequent reduction steps. As shown in Figure S13B, the transformation barrier of the first ∗H_2_O molecule to ∗OH on the Cu_x_O/ZnO surface is 0.47 eV. However, on the Cu_x_/ZnO@De surface, this transformation is barrierless as the calculated energy barrier is negative. Moreover, the barrier for ∗O_2_ desorption on the Cu_x_/ZnO@De surface (4.71 eV) is smaller than on the Cu_x_/ZnO surface (5.23 eV). Therefore, first-principles calculations show that defects in the Cu_x_O/ZnO nanocomposites could facilitate water decomposition and carbon dioxide reduction processes by lowering the reaction energy barriers for intermediate transformation.

The designed band structure that facilitated effective charge transfer between the different components ensured highly efficient charge separation within the composite photocatalysts. Our results suggest that the introduction of Cu_x_O results in an enhanced photo-response among all components, accompanied by significantly improved visible light absorption and utilization capacities. Importantly, by switching between the dominant photo-induced carriers, and manipulating their transport, material performance in various photocatalytic applications could be substantially enhanced, as shown in Figure 5D.

### Limitations of the study

1. Difference between experimental conditions and actual application environment. Although the experimental results show the high performance of the as-prepared nanocomposites in photocatalytic applications, there is insufficient research on the performance and stability of the materials in complex and changing real-world environments. For example, in actual wastewater treatment, the water may contain a variety of complex ions (e.g., heavy metal ions, acid radical ions, etc.), which may react chemically with the hetero-structured nanoparticles, resulting in the structure being disrupted or its performance being degraded.
2. Translation from laboratory scale to large-scale industrial production may be difficult. The current synthesis routes may be suitable for small batch experimental studies. In large-scale preparation, how to ensure homogeneous mixing of precursors, consistency of the reaction, and high-quality collection of the product are all issues to be considered.

## Resource availability

### Lead contact

Requests for further information should be directed to the lead contact, Ming Xu (*hsuming_2001@aliyun.com*).

### Materials availability

This study did not generate new materials.

### Data and code availability

• All data can be obtained from the lead contact, provided the request is reasonable.
• This paper does not report original code.
• Any additional information required to reanalyze the data reported in this paper is available from the lead contact upon request.

## Acknowledgments

This work is supported by 10.13039/501100011243National Lab of Solid State Microstructures, Nanjing University (grant no. M37081), China. We thank Professor Kostya (Ken) Ostrikov for his help in writing and reviewing the manuscript, and LetPub (www.letpub.com) for its linguistic assistance during the preparation of this manuscript.

## Author contributions

H.L.: conceptualization, methodology, investigation, and writing – original draft. K.L.: conceptualization, methodology, validation, and writing – review and editing. Y.D.: DFT calculations and analysis. Q.Z.: software, resources, and writing – review and editing. H.Y.: software and resources. S.X.: resources and writing – review and editing. M.X.: resources, writing – review and editing, supervision, project administration, and funding acquisition.

## Declaration of interests

The authors declare no competing interests.

## STAR★Methods

### Key resources table

**Table:** 

REAGENT or RESOURCE	SOURCE	IDENTIFIER
**Chemicals, peptides, and recombinant proteins**		

Cupric acetate	Mrck & Co. lnc.	61148
Zinc acetate	ChengDu Chron Chemicals Co,.Ltd.	05.001.1822
Zinc nitrate	ChengDu Chron Chemicals Co,.Ltd.	05.001.1868
Silver nitrate	ChengDu Chron Chemicals Co,.Ltd.	05.001.3225
Diethanolamine	ChengDu Chron Chemicals Co,.Ltd.	05.001.3093
Glucose	ChengDu Chron Chemicals Co,.Ltd.	05.006.0289
Tartaric acid	ChengDu Chron Chemicals Co,.Ltd.	05.001.0807
Acrylamide	ChengDu Chron Chemicals Co,.Ltd.	05.001.0644
N, N′-methylene bisacrylamide	ChengDu Chron Chemicals Co,.Ltd.	05.001.0143
Methylene blue	ChengDu Chron Chemicals Co,.Ltd.	05.014.0058
Tetrabutyl titanate	Thermo Fisher Scientific Inc.	22319
Tin Tetrachloride	Thermo Fisher Scientific Inc.	22369
Zirconium dinitrate oxide hydrate	Thermo Fisher Scientific Inc.	043224
Benzoquinone	Chengdu Lingliu Biological Technology Co., Ltd.	TG115626
Ethylenediaminetetraacetic acid disodium salt	Chengdu Lingliu Biological Technology Co., Ltd.	P248680
Ethyl alcohol	Chengdu Lingliu Biological Technology Co., Ltd.	T12091
Isopropanol	Chengdu Lingliu Biological Technology Co., Ltd.	T11788

**Software and algorithms**		

Origin	OriginLab Corporation	https://www.originlab.com
Vienna Ab-initio Simulation Package	University of Vienna	https://www.vasp.at

**Other**		

FA series electronic balance	Shanghai Fangrui Instrument Co., Ltd.	http://www.shfangrui.com
PCJ integrated ultrapure water machine	Chengdu Pincheng Science and Technology Co., Ltd.	http://www.cdpckj.cn
DF-101S thermostatic magnetic stirrer	Gongyi Yuhua Instrument Co., Ltd.	http://www.gyyuhua.com
101 electric blast drying oven	Beijing Zhongxingweiye Instrument Co., Ltd	http://www.zhongxingwy.cn
MF-1100C-L muffle furnace	Anhui Best Equipment and Technology Co., Ltd.	http://www.ahbeq.com
PLS-SXE300D/300DUV Xenon lamp source	Beijing Perfectlight Technology Co., Ltd.	https://www.perfectlight.cn
V-1100D spectrophotometer	Shanghai Mapada Instruments Co., Ltd.	http://www.mapada.com.cn
FZ-A light irradiation meter	Beijing Shida Photoelecctric Technology Co., Ltd.	http://www.peifbnu.com
KW-4C spin coater	Beijing Setcas Electronics Co., Ltd.	https://setcas.com
MFC 300 mass flow controller	Suzhou Aitoly Electronic Equipment Co., Ltd.	https://www.aitoly.com
Keithley 2700 data acquisition/switching system	Guangzhou Mitek Data Technology Co., Ltd.	http://www.gzmitek.com

### Experimental model and study participant details

There are no experimental models (animals, human participants, plants, microbe strains, cell lines, primary cell cultures) used in the study.

### Method details

#### Material synthesis

##### Preparation of Cu_x_O nano-particles

Cu_x_O NPs were prepared through a mild sol-gel method. Typically, precursor cupric acetate (0.02 mol) and precipitant diethanolamine (C_4_H_11_NO_2_, 2 mL) were dispersed into 60 mL ethyl alcohol, followed by mixing and stirring in a water bath at 60°C for 2 h, and a stable clear sol system formed. After standing for 48 h, the resultant gel was dried at 80°C and then calcined at 400°C for 10 h, Cu_x_O nano-particles were obtained.

##### Method for the sol-gel preparation of Cu_x_O/MOS composite nano-particle photocatalysts

Taking Cu_x_O/ZnO for instance, the as-synthesized Cu_x_O, zinc acetate (0.02 mol), and diethanolamine (2 mL) were dispersed in 60 mL ethanol, then heated at 60°C in a water bath and stirred with a magnetic stirrer for 2 h. After static placing for 48 h, the gel was dried at 80°C and then calcined at 450°C for 10 h, sol-gel-based Cu_x_O/ZnO NPs were successfully fabricated, labeled as 3%CXZ, 5%CXZ, and 10%CXZ to reflect the Cu_x_O amount. The required reagents of Ti, Sn, and Zr precursors for the preparation of Cu_x_O/TiO_2_, Cu_x_O/SnO_2_, and Cu_x_O/ZrO_2_ are depicted in key resources table.

##### Method for the preparation of polymer network structure based Cu_x_O/MOS composite nano-particle photocatalysts

All the polymer network gel products are denoted with “(r)” to represent the re-design of the major surface carrier type. Taking Cu_x_O/ZnO(r) for instance, the as-synthesized Cu_x_O, precursor zinc nitrate hexahydrate (Zn(NO_3_)_2_·6H_2_O, 0.02 mol), precipitant diethanolamine (C_4_H_11_NO_2_, 2 mL), glucose (12 g), complexing agent tartaric acid (C_4_H_6_O_6_, 0.03 mol), crosslinker acrylamide (C_3_H_5_NO, 0.15 mol) and monomer N, N′-methylene bisacrylamide (C_7_H_10_N_2_O_2_, 0.03mol) were dispersed into 60 mL of deionized water, followed by stirring in a water bath at 90°C to obtain 3-day network wet gel. The wet gel was dried at 120°C, and Cu_x_O/ZnO NPs were finally obtained by calcining the resultant xerogel at 650°C for 10 h, which was the optimal annealing temperature for balancing the surface specific area and crystallinity of the photocatalysts. Diethanolamine here acted as a PH buffer and provided a reducing atmosphere, and glucose prevents the gel from rapidly contracting during the period of temperature rise. Likewise, the photocatalysts were labeled as 3%CXZ(r), 5%CXZ(r), and 10%CXZ(r) to reflect the Cu_x_O amount.

#### Material characterization

The crystalline micro-structure studies were carried out in a DX-2000 powder XRD instrument, the incident X-ray energy was measured as 3 kV with an incident angle ranging from 20° to 80°. A JEOL JSM-7500F c-FEG scanning electron microscope (SEM) operated at 15 keV was used to observe the surface morphology of the nanocrystals. Transmission Electron Microscope (TEM) and High-resolution TEM (HRTEM) were performed on an FEI (Thermo Fisher) Tecnai G2 F20 microscope operated at 200 kV acceleration voltage, the nominal point resolution was 0.19 nm under the ultra-high-resolution mode, which was ideal for distinguishing the lattice planes of Cu_x_O and ZnO. The electron paramagnetic resonance (EPR) spectra were performed on a Bruker A300 spectrometer at room temperature. The absorption capacities of the materials were characterized by a 319.2 nm incident wavelength UV-vis spectroscopy (UV-vis, UV-2550) with an integral sphere. The surface photovoltaic effects were detected by an instrument (Jilin University) consisting of a source of monochromatic light, a lock-in amplifier (SR830-DSP) with a light chopper (SR540), and a photovoltaic cell at room temperature. Time-resolved photoluminescence measurements were carried out in an Edinburgh FLS1000 transient fluorescence spectrometer. The steady-emission properties were evaluated by a PerkinElmer FL-8500 photoluminescence spectroscopy. The *in-situ* photoluminescence characterization was also performed in a PerkinElmer 8500F photoluminescence spectroscopy. For ultraviolet photoelectron spectroscopy (UPS) the samples were drop casted onto ITO glasses (1 × 1 cm) and analyses on the Escalab 250xi instrument using He I-UV source (21.22 eV). An excitation system was constructed using a 150 W xenon lamp with a dispersion grating and a 150 μm optical slit that selects a single monochromatic illumination wavelength in the 200 nm–900 nm spectral range. The 150 μm incident slit with a grating coupling system provides a spectral range of up to 200 nm–900 nm with a wavelength resolution of approximately 10 nm. Each spectral acquisition duration was 1.5 min to obtain an *in situ* spectral series. Sample/MB suspension (Sample: 0.05g; MB: 4 mg L^−1^, 100 mL) was prepared using Kunshan Ultrasound KQ3200DE. The surface chemistry states were measured by a VG Scientific ESCALAB 210 X-ray photoelectron spectroscopy (XPS) with a Mg K X-ray source, all the binding energies were calibrated for the C1 s peak at 285.0 eV.

#### Photocatalytic testing

##### Photocatalytic degradation

The photocatalytic degradation properties of Cu_x_O/MOSs heterostructures were investigated by considering the degradation of methylene blue (MB) under simulate sunlight illumination. The initial absorbance (A_0_) scanning of the dye (4 mg L^−1^) solution was carried out at the characteristic wavelength of 664 nm. Typically, 50 mg of powder was dispersed in 100 mL MB solution. After 10 min of ultrasonic oscillation, the sample/dye suspension was left in a dark environment and stood for 20 min to achieve adsorption-desorption equilibrium. The solution with photocatalyst was then irradiated using a xenon lamp, the irradiation distance was 20 cm, and the irradiation intensity was 0.17 W cm^−2^ 5 mL of the residual contamination solution was taken out at regular intervals and was centrifuged at a speed of 6000 r·min^−1^ using a centrifugal machine, then the supernatant was collected and the absorbance (A_t_) was measured by spectrophotometer.

The relations between real-time degradation rate (Y) and kinetic degradation constants (K) are shown in Equations 1 and 2:

(Equation 1) $$Y=\left(C_{0\;}-C_{t}\right)/C_{0}\times 100\%=\left(A_{0\;}-A_{t}\right)/A_{0}\times 100\%$$

(Equation 2) $$Kt=\ln\left(C_{0\;}-C_{t}\right)$$

Where C_0_ is the initial concentration of MB solution and C_t_ represents the measured concentration.

##### Photocatalytic H_2_ evolution

Photocatalytic hydrogen evolution experiments were performed using a PerfectLight, Labsolar-IIIAG photocatalytic system (Beijing Perfectlight Science & Technology Co., Ltd, China). The light source was a 300 W Xe lamp. The photocatalytic reaction was conducted in a 250 mL sealed quartz reactor. Typically, 50 mg photocatalyst was put in a mixture of 50 mL deionized water, 50 mL methanol, and 15 mL trolamine (scavengers), with ultrasonic dispersion for 10 min. Afterward, the photocatalytic system was degassed for 20 min to remove air completely. The sample was irradiated for 4 h in a closed water circulating system at 25°C. The amount of H_2_ evolved was detected using an online TCD gas chromatograph (Tech, GC-9700, nitrogen gas carrier) at intervals of 1 h.

##### Photocatalytic CO_2_ reduction

Photocatalytic CO_2_ reduction experiments were carried out in a homemade 500 mL closed gas system (Heilongjiang University). Typically, 50 mg photocatalyst powder were dispersed in 5 mL of deionized water. The apparatus was initially vacuumed and then pumped with high-purity CO_2_ at 0.08 MPa. The sample was irradiated for 4 h with a 300 W xenon lamp, the reaction temperature was kept at about 5°C. The amount of CO and CH_4_ produced was determined by a gas chromatograph (GC-2014 with FID detector; Shimadzu Corp., Japan) at intervals of 1 h.

##### Gas-sensing performance evaluation

500-nm-thick silicon dioxide (SiO_2_) passivated n-type Si (100) wafers were used to serve as substrates. A Ti (200-nm-thick)/Au (500-nm-thick) double-layer electrode was deposited on the surface of SiO_2_ by thermal evaporation treatment. Then the interdigital electrodes with a finger width of 50 μm and gap width of 50 μm were patterned by conventional photolithography and lift-off process. The sensors were fabricated by a tractable spin-coating process. Firstly, the as-prepared Cu_x_O/MOS nanocrystals were dissolved in ethanol to achieve a concentration of 3.0 mg mL^−1^. Secondly, the sample suspension was spin-coated onto the interdigital electrodes at 500 rpm for 6 s and 3000 rpm for 30 s, respectively, followed by drying at 80°C for 8 h. Gas-sensitive performance was measured with an instrument consisting of a test chamber equipped with UV-LED light sources (365 nm, 3 W), mass flow controller (MFC300, Suzhou Aitoly Electronic Equipment Co., Ltd., Suzhou, China), and Keithley 2700 source meter (Guangzhou Mitek Data Technology Co., Ltd., Guangzhou, China). All experimental results were obtained at room temperature (298.15 K). During NO_2_ sensing tests, the sensors were fixed into a little sealed metal chamber and then purged by dry air to avoid the sensors’ performance being impacted by ambient humidity and the distance between the sensor and UV-LED was 15 mm. The real-time resistance (denoted as R) of the sensors was obtained by Keithley 2700 source meter/Data Acquisition System at a constant flow rate of 200 mL min^−1^ when NO_2_ concentration changed from 1 to 5 ppm.

#### First-principles atomistic simulations

All spin-polarized density functional theory (DFT) calculations were implemented within the Vienna *ab initio* Simulation Package (VASP). The Perdew Burke Ernzerhof (PBE) functional and the generalized gradient approximation (GGA) approach were used to describe the electron exchange correlation potential. The projector-augmented-wave (PAW) pseudopotential was applied to treat the ion-electron interactions. The plane-wave cutoff energy was set to 600 eV and with a 5 × 5×1 Monkhorst−Pack k-point mesh was applied to sample the Brillouin zone in the electronic and ionic optimization, until an energy convergence of 10^−5^ eV/atom and a force convergence of −0.02 eV/Å were achieved for each atom according to the conjugate gradient (CG) algorithm. To describe the van der Waals interactions between the substrate and adsorbates, Grimme’s semiempirical DFT-D3 approach was used to correct Gibbs free energy. Furthermore, to avoid the interactions between different interlayers which were produced from the periodic boundary condition, a 20 Å vacuum space was set along the z direction. To gain insights into the electronic structure, a denser 11 × 11×1 Monkhorst−Pack grid was used in the density of states and *d*-band center calculations. The computational hydrogen electrode (CHE) model was used to correct the Gibbs free energy under ambient conditions. The CHE was defined as the free energy change of a proton and electron pair transfer.

### Quantification and statistical analysis

Statistical analysis of data was performed using Excel (Microsoft) and Origin (OriginLab).

## Supplemental information

Document S1. Figures S1–S13, Tables S1, and S2

Data S1. Raw data of relevant figures, related to the main manuscript and supplemental information
