# Supplementary material for: Controllable surface carrier type of metal oxide nanocrystals for multifunctional photocatalysis
Source: iScience. 2025 Jan 4;28(2):111750. doi: 10.1016/j.isci.2025.111750 (PMC11787535; doi:10.1016/j.isci.2025.111750)
Supplement: Document S1. Figures S1–S13, Tables S1, and S2 [file mmc1.pdf]

iScience, Volume *28*

Supplemental information

Controllable surface carrier type of metal oxide nanocrystals for multifunctional photocatalysis

Han Li, Yingchun Ding, Kaiyi Luo, Qiuping Zhang, Huan Yuan, Shuyan Xu, and Ming Xu

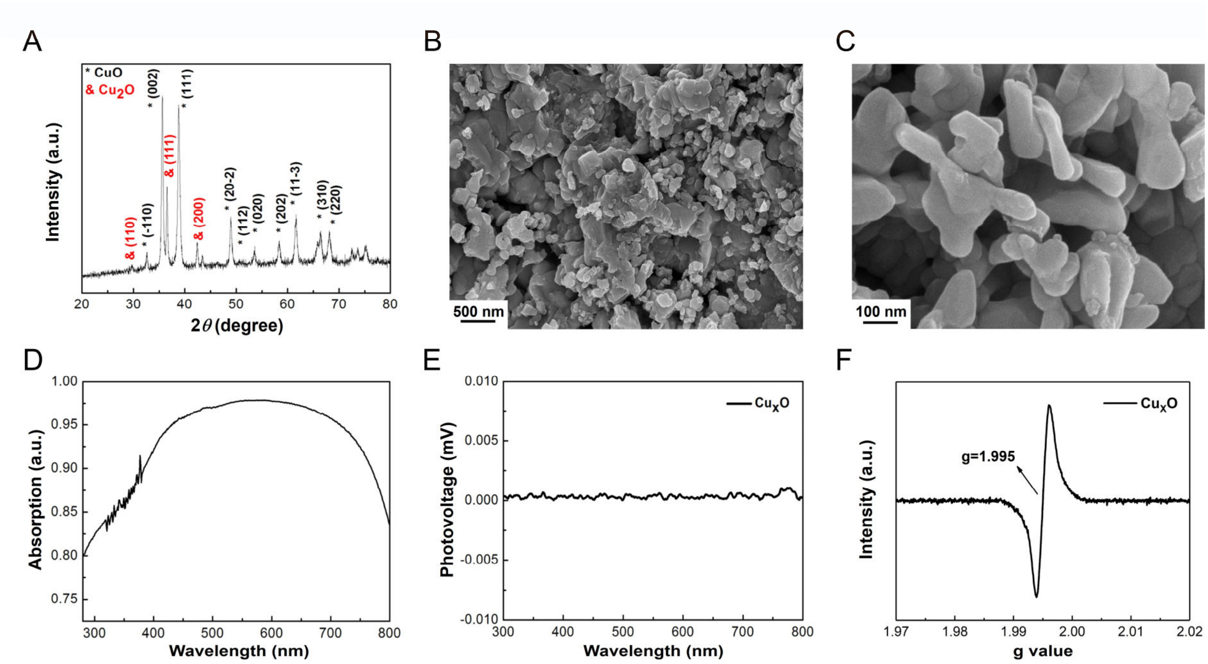

Figure S1. Characterization of Cu_xO. (A) XRD pattern. (B) SEM image. (C) TEM image. (D) UV–vis absorption spectrum. (E) Steady-state SPV pattern. (F) EPR spectrum of copper vacancies. Related to Figure 2 and Figure 3.

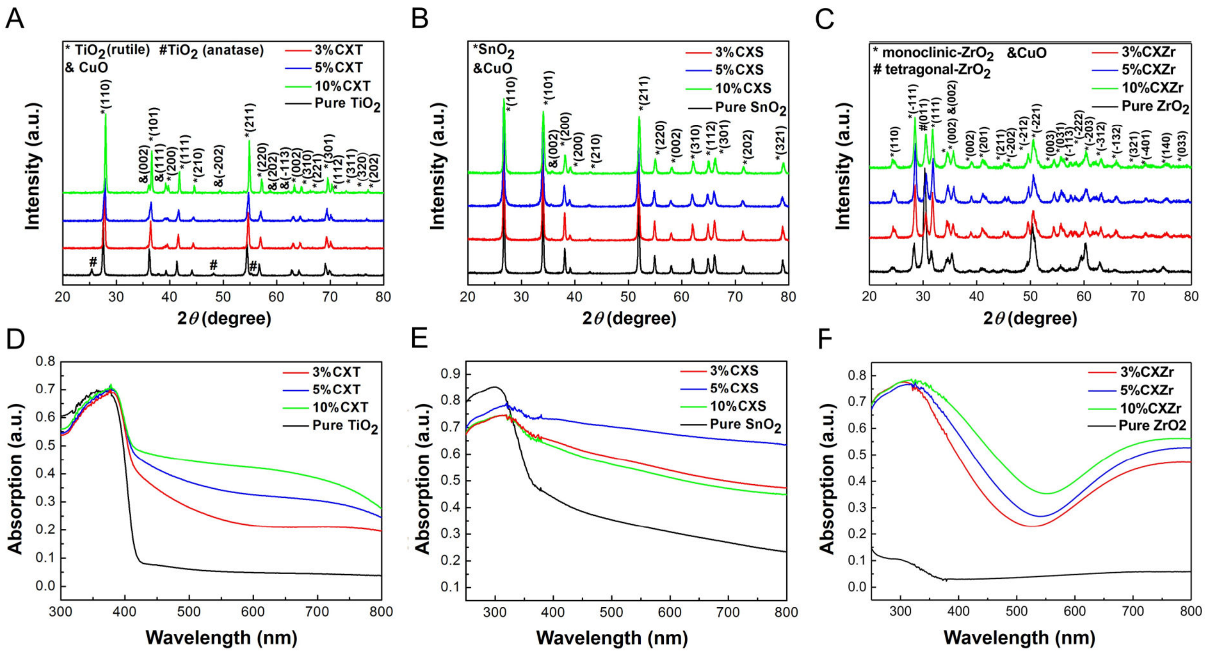

Figure S2. XRD patterns and UV-vis absorption spectra of the Cu_xO/MOSs samples. XRD patterns of (A) Cu_xO/TiO_2, (B) Cu_xO/SnO_2, and (C) Cu_xO/ZrO_2. UV-vis absorption spectra of (D) Cu_xO/TiO_2, (E) Cu_xO/SnO_2, and (F) Cu_xO/ZrO_2. Related to Figure 2 and Figure 3.

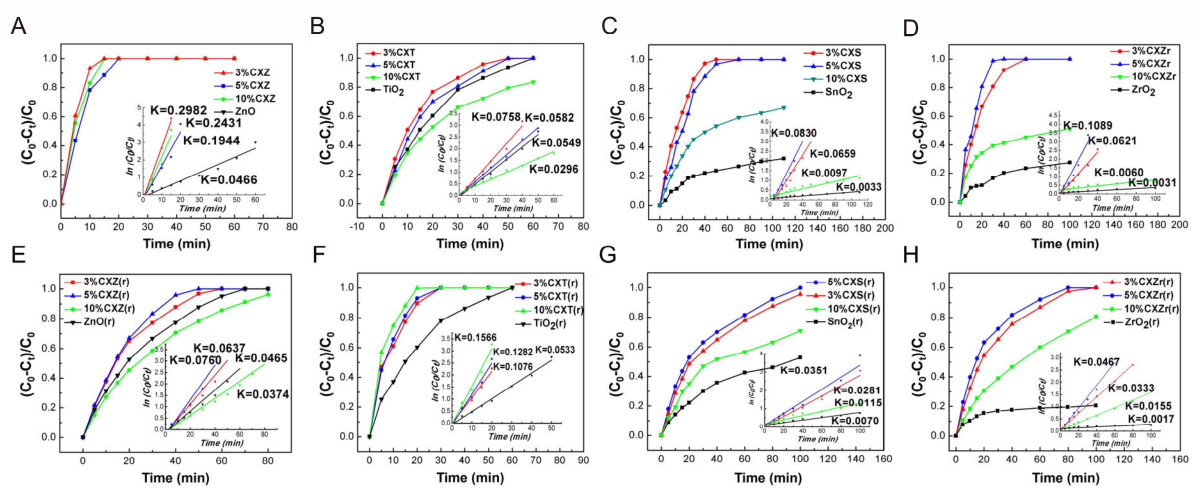

Figure S3. Photocatalytic MB degradation curves for the Cu_xO/MOSs and Cu_xO/MOSs(r) samples under simulated sunlight irradiation. (A) Cu_xO/ZnO. (B) Cu_xO/TiO_2. (C) Cu_xO/SnO_2. (D) Cu_xO/ZrO_2. (E) Cu_xO/ZnO(r). (F) Cu_xO/TiO_2(r). (G) Cu_xO/SnO_2(r). (H) Cu_xO/ZrO_2(r). Related to Figure 4.

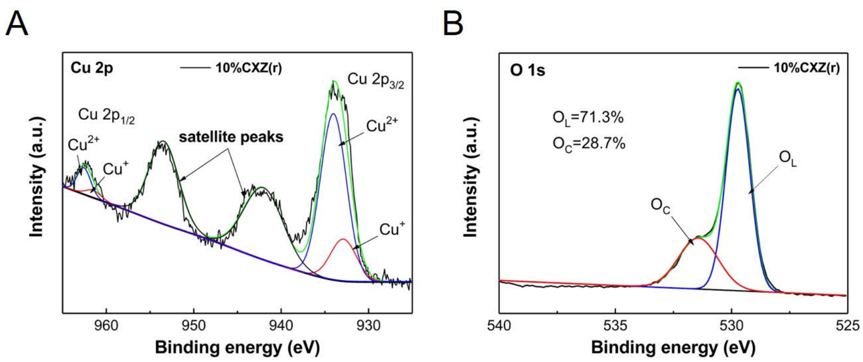

Figure S4. XPS spectra of re-engineered CXZ(r) samples. High-resolution (A) Cu 2p and (B) O 1s spectra. Related to Figure 2.

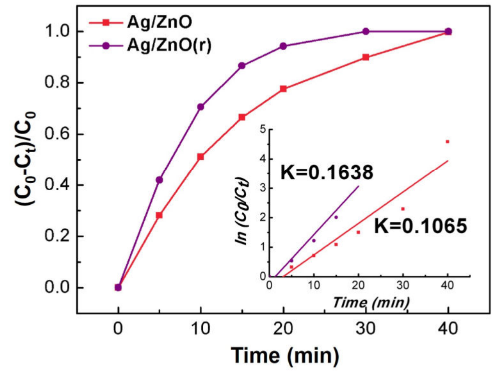

Figure S5. Photocatalytic MB degradation with Ag-decorated samples. Related to Figure 4.

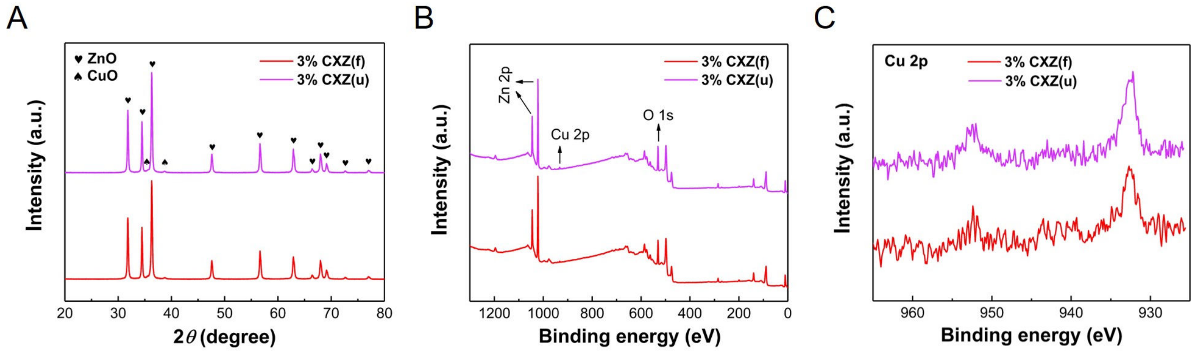

Figure S6. Stability testing of 3%CXZ during photocatalytic process. (A) Comparison of XRD patterns. (B) Comparison of XPS survey. (C) Comparison of high-resolution Cu 2p spectra. Related to Figure 4.

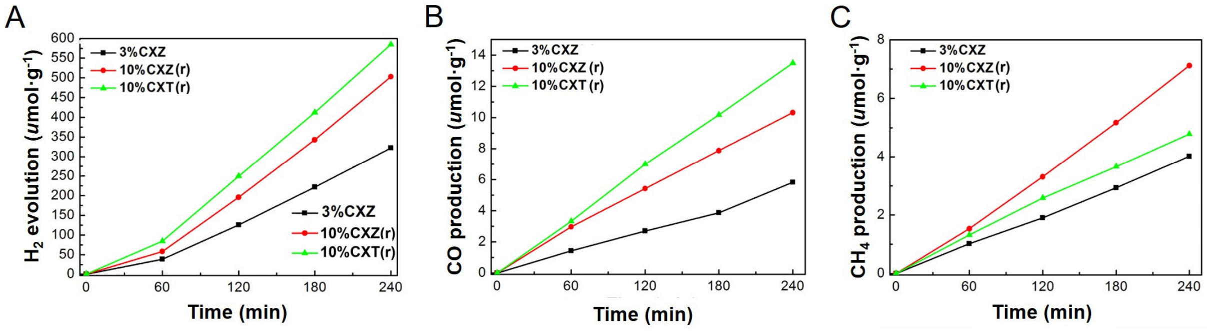

Figure S7. Photocatalytic reduction performance of CXZ and CXZ(r) samples. (A) H_2 evolution curves. (B and C) CO_2 reduction curves. Related to Figure 4.

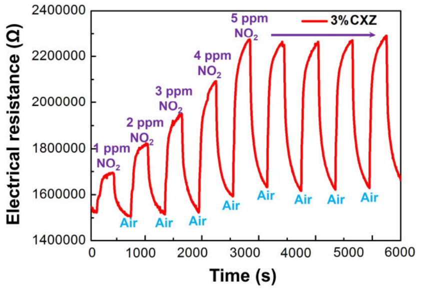

Figure S8. Gas-sensing curves for 3%CXZ sample. Related to Figure 4.

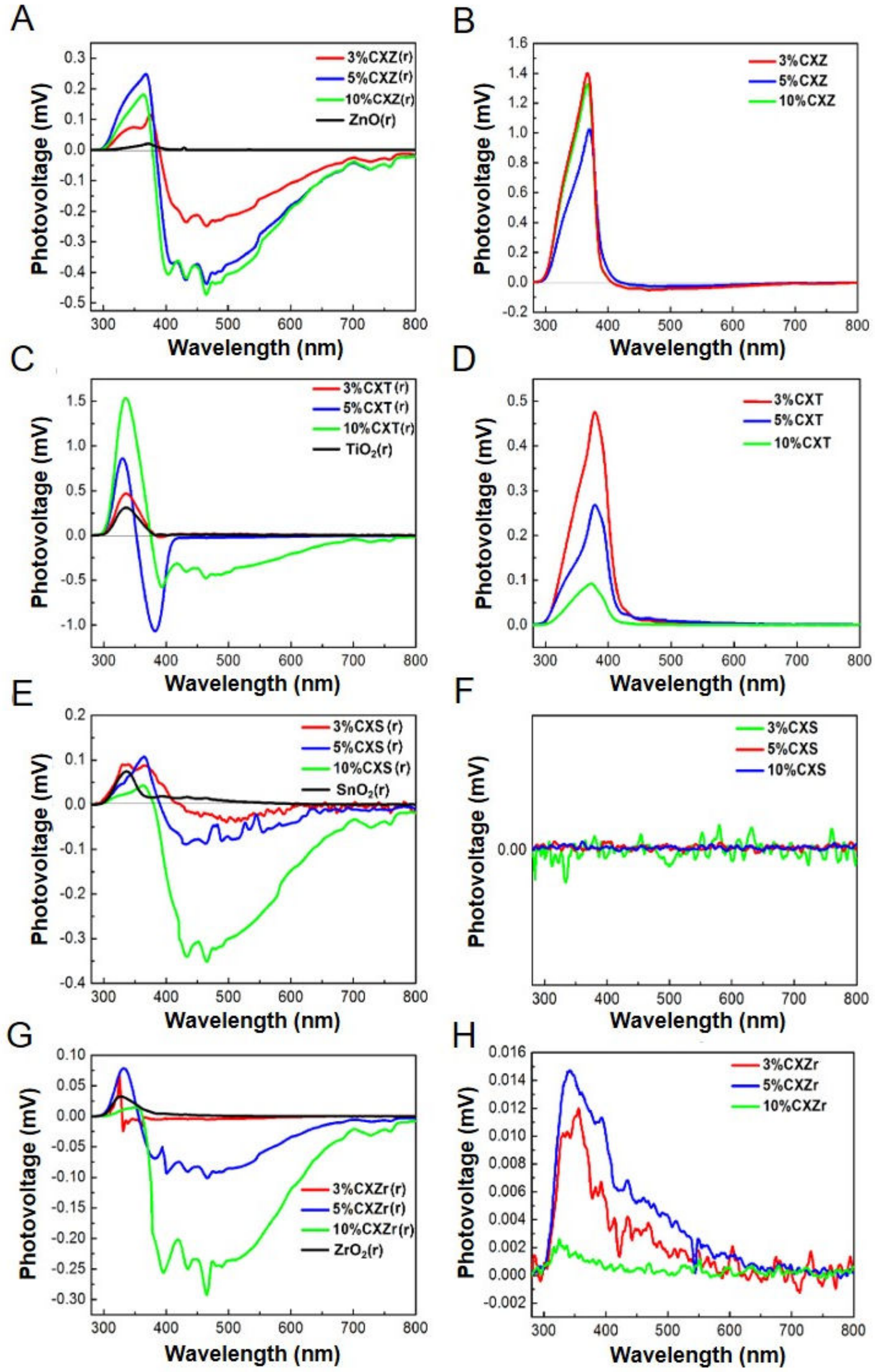

Figure S9. Steady-state SPV results of Cu_xO/MOSs and re-engineered Cu_xO/MOSs (Cu_xO/MOS(r)) samples. (A and B) Re-engineered Cu_xO/ZnO and Cu_xO/ZnO. (C and D) Re-engineered Cu_xO/TiO_2 and Cu_xO/TiO_2. (E and F) Re-engineered Cu_xO/SnO_2 and Cu_xO/SnO_2. (G and H) Re-engineered Cu_xO/ZrO_2 and Cu_xO/ZrO_2. Related to Figure 4.

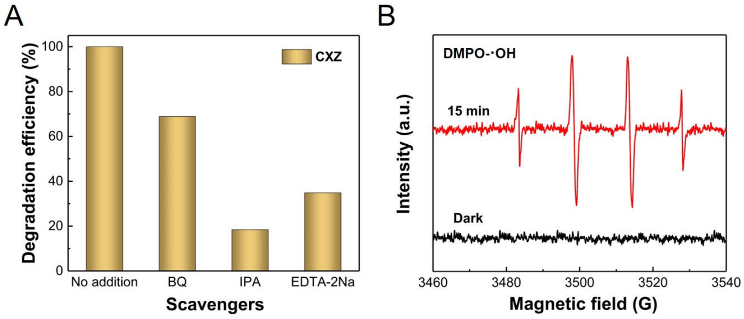

Figure S10. Active species detection during MB degradation with 3%CXZ. (A) Active species capturing experiments. (B) EPR analysis of hydroxyl radicals (·OH). Related to Figure 5.

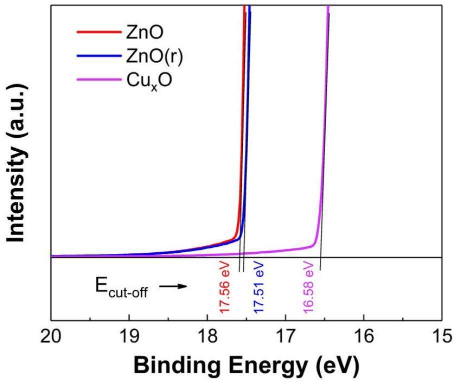

Figure S11. UPS analysis of ZnO, ZnO(r), and Cu_xO. Related to Figure 5.

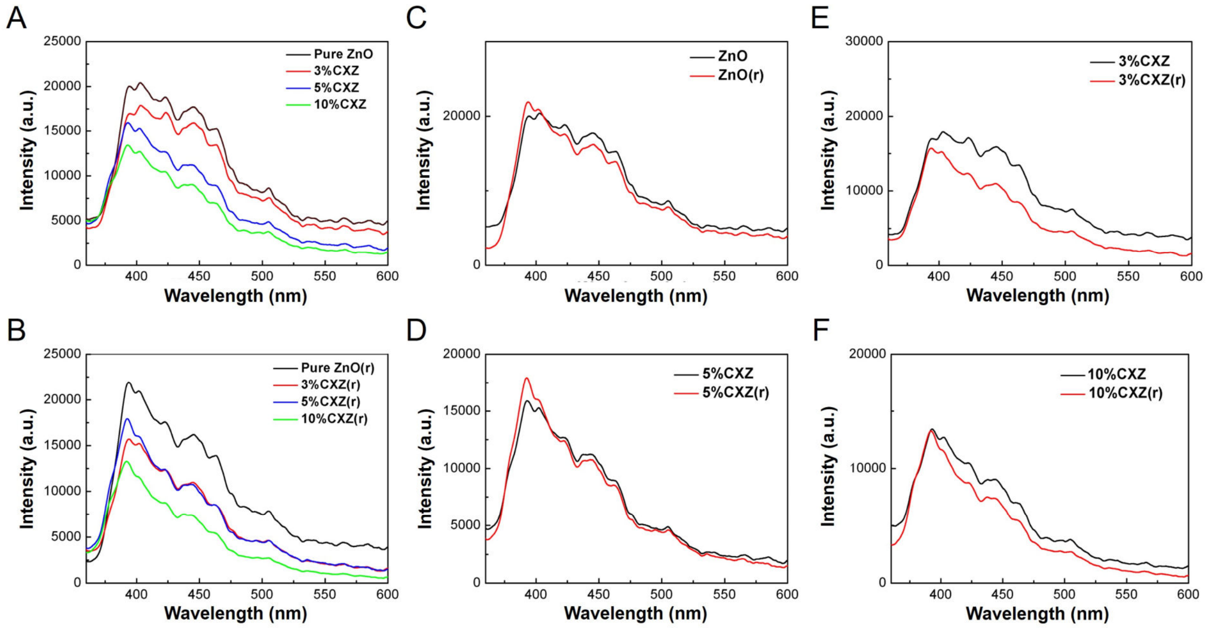

Figure S12. Steady-state PL spectra of CXZ and CXZ(r) samples. PL spectra of (A) CXZ and (B) CXZ(r) samples. (C) Comparison of PL spectra between ZnO and ZnO(r). (D) Comparison of PL spectra between 5%CXZ and 5%CXZ(r). (E) Comparison of PL spectra between 3%CXZ and 3%CXZ(r). (F) Comparison of PL spectra between 10%CXZ and 10%CXZ(r). Related to Figure 5.

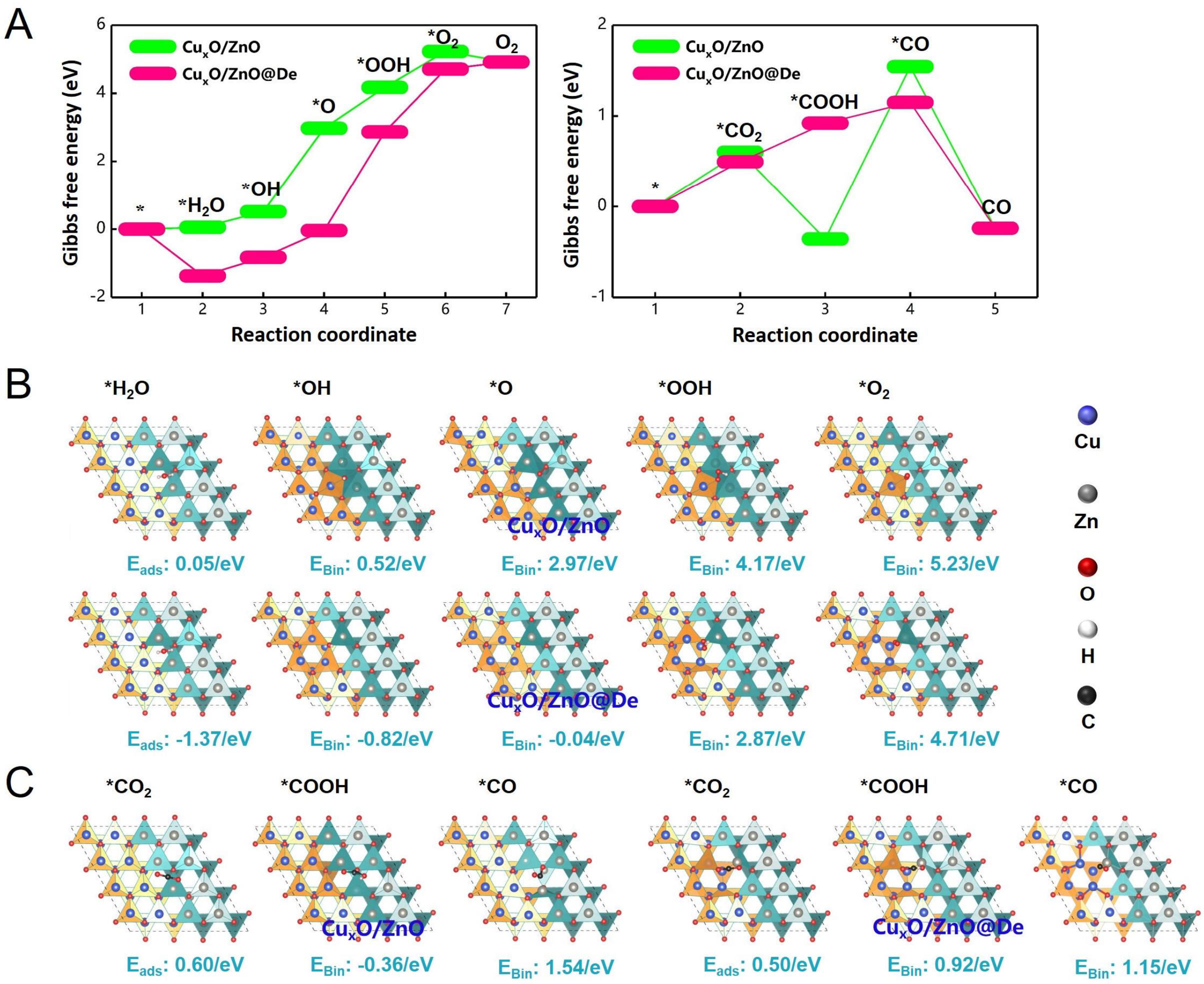

Figure S13. First principles calculations. (A) Gibbs free energy maps of the photocatalytic water splitting (left) and CO_2 reduction (right) process on the surface of the pure Cu_xO/ZnO and defect-rich Cu_xO/ZnO ($Cu_xO/ZnO@De$) nanocomposites, where * denotes the intermediate state. Reaction pathways of (B) complete water decomposition and (C) CO_2 reduction circles on the surface of Cu_xO/ZnO and $Cu_xO/ZnO@De$. Related to STAR Methods.

Table S1. The microstructure parameters calculated according to XRD for Cu_xO/ZnO samples. Related to Figure 2.

Samples	2θ	d (100)	d(002)	d(101)	a	c	D	D (CuO)	V (Å3)
ZnO	31.88	0.2805	0.2594	0.2469	0.3239	0.5188	32.35	-	47.13
3%CXZ	31.93	0.2801	0.2592	0.2465	0.3234	0.5184	31.5	21.37	46.95
5%CXZ	32.01	0.2794	0.2586	0.2461	0.3226	0.5199	24.22	28.48	46.85
10%CXZ	32.08	0.2787	0.2580	0.2455	0.3218	0.5160	23.75	32.04	46.27

Table S2. Comparison of degradation, hydrogen evolution, and reduction performance of Cu_xO/MOS and Cu_xO/MOS (r) with literatures. Related to Figure 4.

Main material	Method	Degradation time (min)			H_2 evolution (mmol·h^{-1}g^{-1})		CO_2 reduction (μmol·h^{-1}g^{-1})		Ref.
TiO_2	hydrogenation	dye	TiO_2	Black TiO_2	TiO_2	Pt/Black TiO_2	–		1
		MB	50	8	–	10.0			
ZnO	solvothermal, air-annealing	–			–		CO		2
							0.36		
ZnO	hydrogenation	dye	ZnO	Black ZnO	–		–		3
		MB	>60	30					
P25	photo-deposition	–			P25	Pt-Cu/TiO_2	CH_4	CO	4
					0.5×10^{-2}	0.7×10^{-1}	5.9	9.8	
TiO_2	surfactant template, wet impregnation	dye	TiO_2	Cu/TiO_2	TiO_2	Cu/TiO_2	–		5
		MO	120	~30	0.6×10^{-2}	1.5×10^{-1}			
g-C_3N_4	calcining urea, sol-hydro-thermal, photo-deposition	–			g-C_3N_4	Ag/TiO_2/g-C_3N_4	CH_4		6
					1.2×10^{-2} ($\lambda > 400$)	1.7×10^{-1} ($\lambda > 400$)	0.45 ($\lambda > 420$)		
Cu_x/ZnO, Cu_x/TiO_2, et al.	polymer network gel, sol-gel	dye	ZnO	3%CXZ(r)	3%CXZ(r)	10%CXT	CH_4	CO	this work
		MB	60	15	0.8×10^{-1} ($\lambda > 400$)	1.5×10^{-1} ($\lambda > 400$)	1.0	3.5	

REFERENCES

1. Chen, X., Liu, L., Yu, P.Y., and Mao, S.S. (2011). Increasing solar absorption for photocatalysis with black hydrogenated titanium dioxide nanocrystals. Science *331*, 746–750. 10.1126/science.1200448.
2. Sayed, M., Xu, F., Kuang, P., Low, J., Wang, S., Zhang, L., and Yu, J. (2021). Sustained CO_2-photoreduction activity and high selectivity over Mn, C-codoped ZnO core-triple shell hollow spheres. Nat. Commun. *12*, 4963. 10.1038/s41467-021-25007-6.
3. Xia, T., Wallenmeyer, P., Anderson, A., Murowchick, J., Liu, L., and Chen, X. (2014). Hydrogenated black ZnO nanoparticles with enhanced photocatalytic performance. RSC Adv. *40*, 41654–41658. 10.1039/c4ra04826a.
4. Zhai, Q., Xie, S., Fan, W., Zhang, Q., Wang, Y., Deng, W., and Wang, Y. (2013). Photocatalytic conversion of carbon dioxide with water into methane: Platinum and Copper(I) oxide co-catalysts with a core-shell structure. Angew. Chemie - Int. Ed. *52*, 5776–5779. 10.1002/anie.201301473.
5. Trofimovaite, R., Parlett, C.M.A., Kumar, S., Frattini, L., Isaacs, M.A., Wilson, K., Olivi, L., Coulson, B., Debgupta, J., Douthwaite, R.E., et al. (2018). Single atom Cu(I) promoted mesoporous titanias for photocatalytic Methyl Orange depollution and H_2 production. Appl. Catal. B Environ. *232*, 501–511. 10.1016/j.apcatb.2018.03.078.
6. Zhang, X., Zhang, X., Li, J., Sun, J., Bian, J., Wang, J., Qu, Y., Yan, R., Qin, C., and Jing, L. (2018). Exceptional visible-light activities of g-C_3N_4 nanosheets dependent on the unexpected synergistic effects of prolonging charge lifetime and catalyzing H_2 evolution with H_2O. Appl. Catal. B Environ. *237*, 50–58. 10.1016/j.apcatb.2018.05.034.
