# Supplementary material for: Controllable surface carrier type of metal oxide nanocrystals for multifunctional photocatalysis
Source: iScience. 2025 Jan 4;28(2):111750. doi: 10.1016/j.isci.2025.111750 (PMC11787535; doi:10.1016/j.isci.2025.111750)
Supplement: Data S1. Raw data of relevant figures, related to the main manuscript and supplemental information [file mmc2.zip › Data/DFT/Newest Data/ZnO.pptx]

## Slide 1
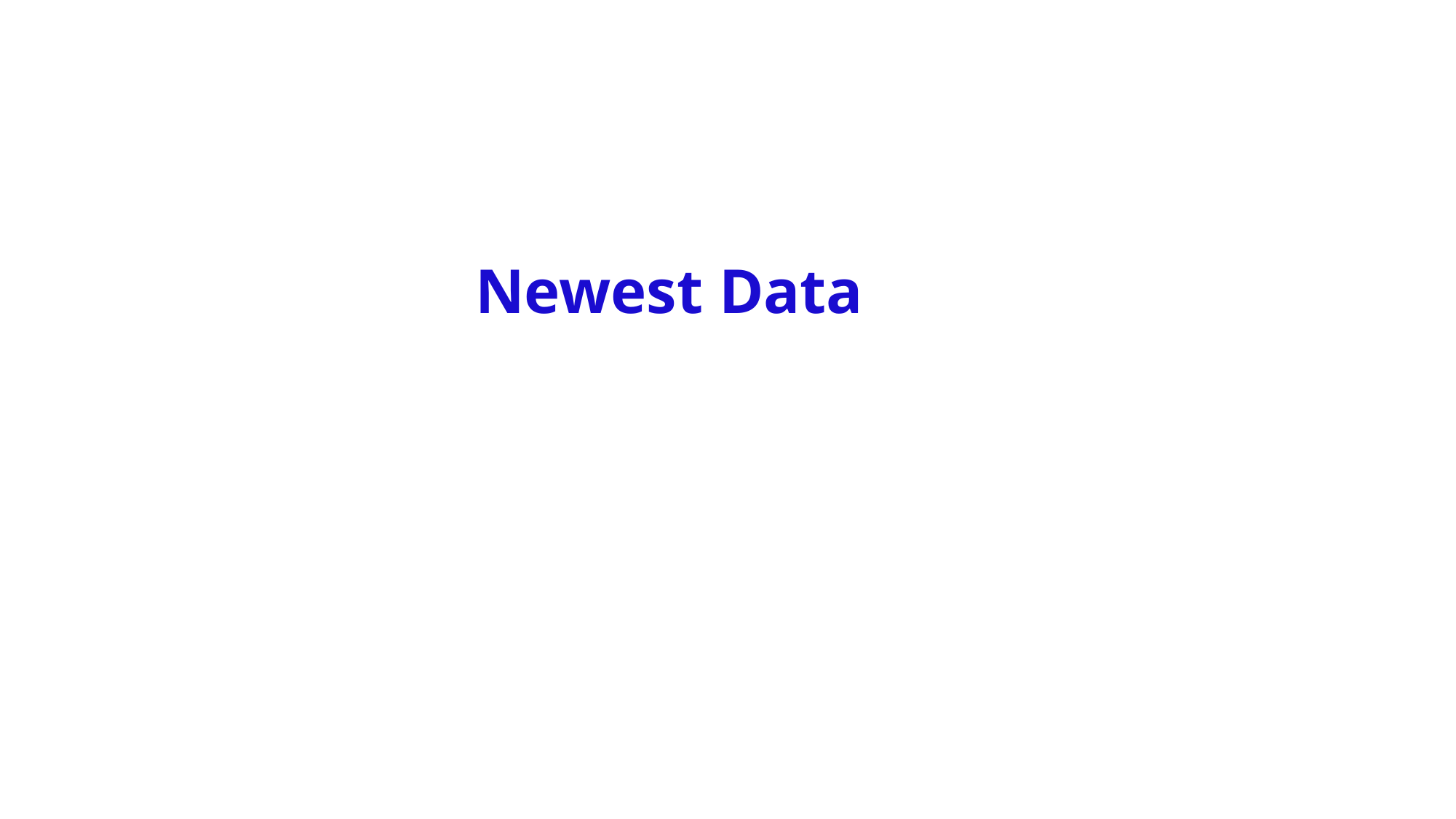

Newest Data

## Slide 2
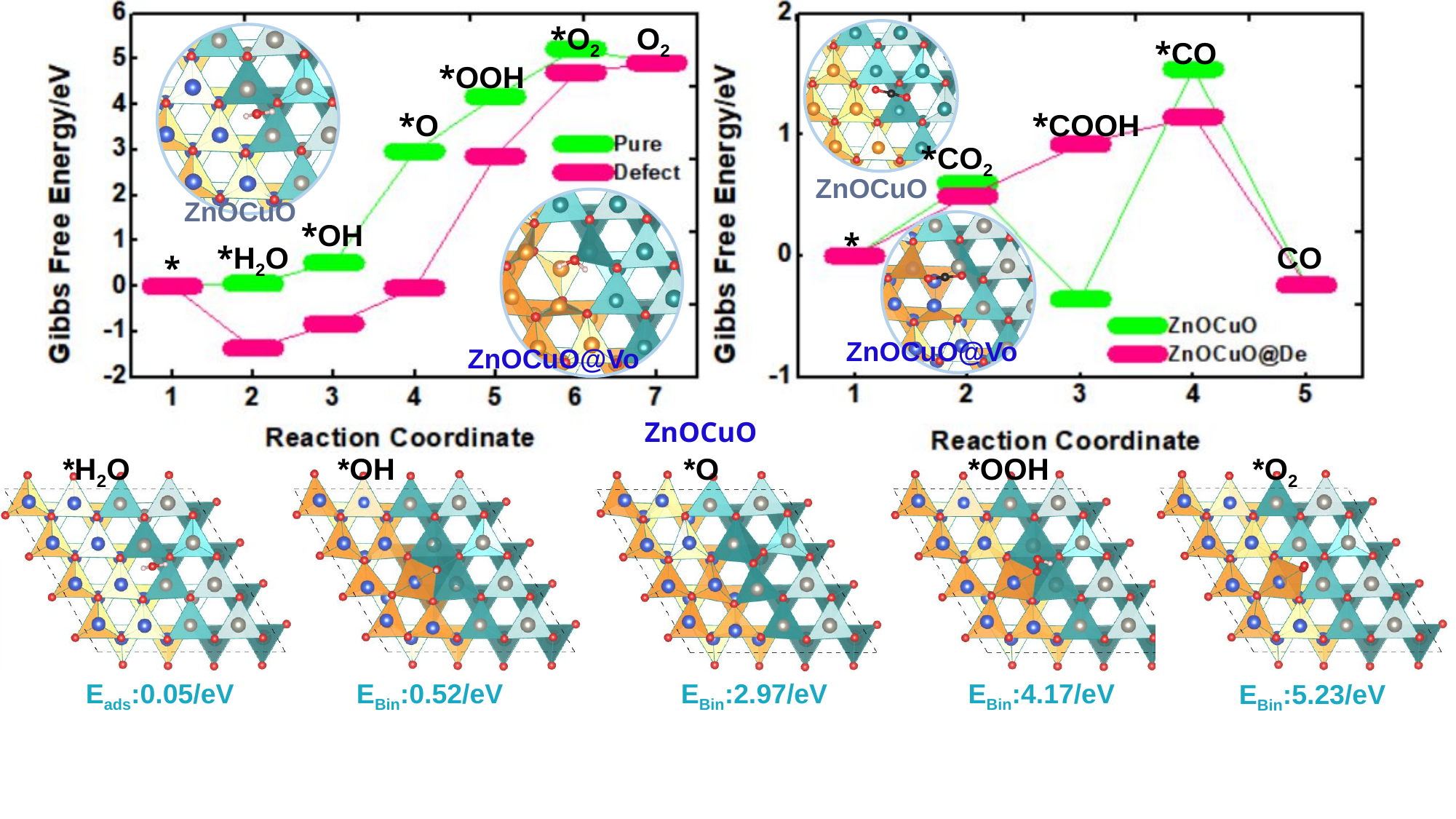

*O2
O2
*CO
*OOH
*O
*COOH
*CO2
ZnOCuO
ZnOCuO
*OH
*
*H2O
CO
*
ZnOCuO@Vo
ZnOCuO@Vo
ZnOCuO
*H2O
*OH
*O
*OOH
*O2
Eads:0.05/eV
EBin:0.52/eV
EBin:2.97/eV
EBin:4.17/eV
EBin:5.23/eV

## Slide 3
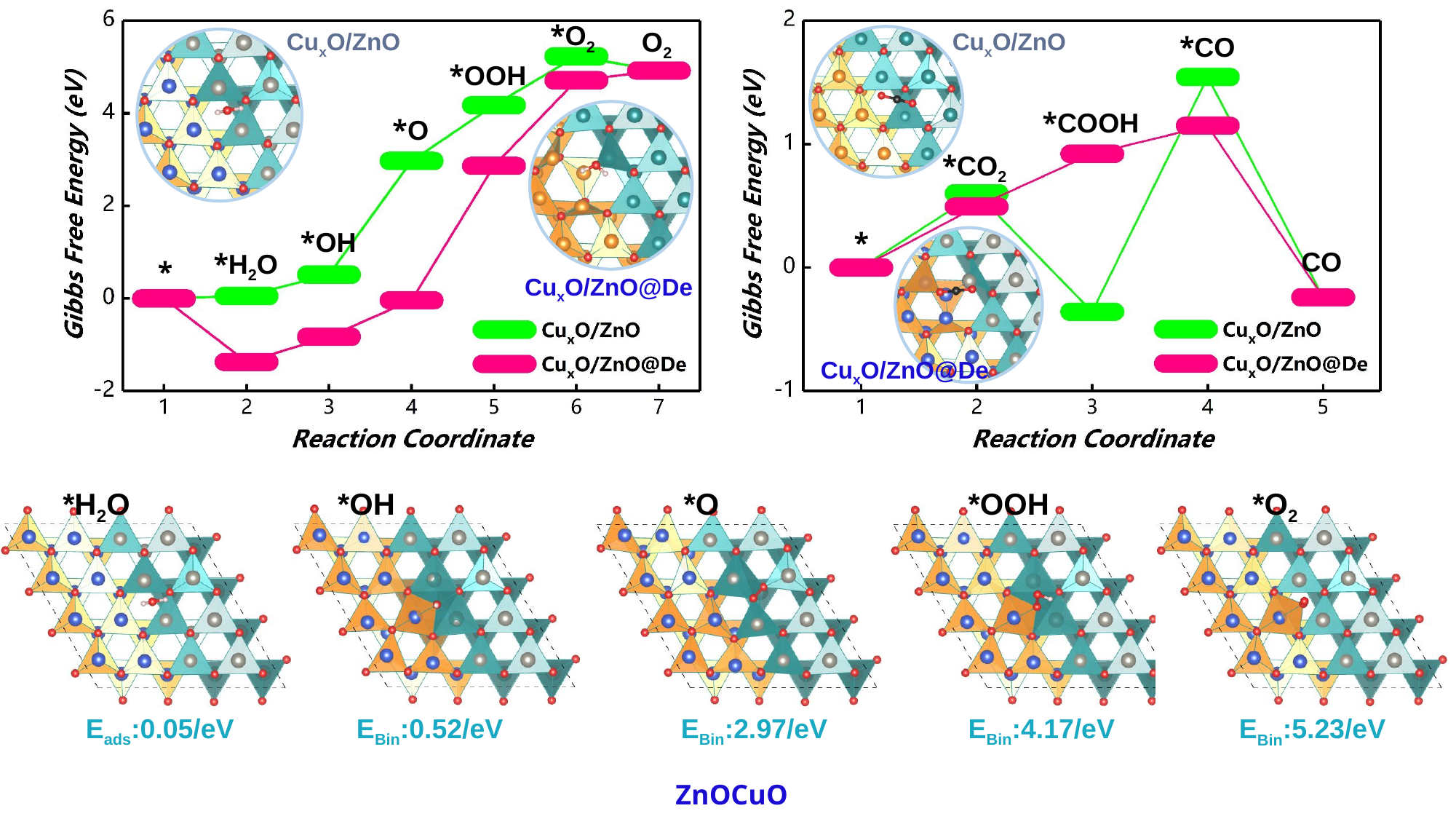

*O2
O2
CuxO/ZnO
CuxO/ZnO
*CO
*OOH
*COOH
*O
*CO2
*OH
*
CO
*H2O
*
CuxO/ZnO@De
CuxO/ZnO@De
*H2O
*OH
*O
*OOH
*O2
Eads:0.05/eV
EBin:0.52/eV
EBin:2.97/eV
EBin:4.17/eV
EBin:5.23/eV
ZnOCuO

## Slide 4
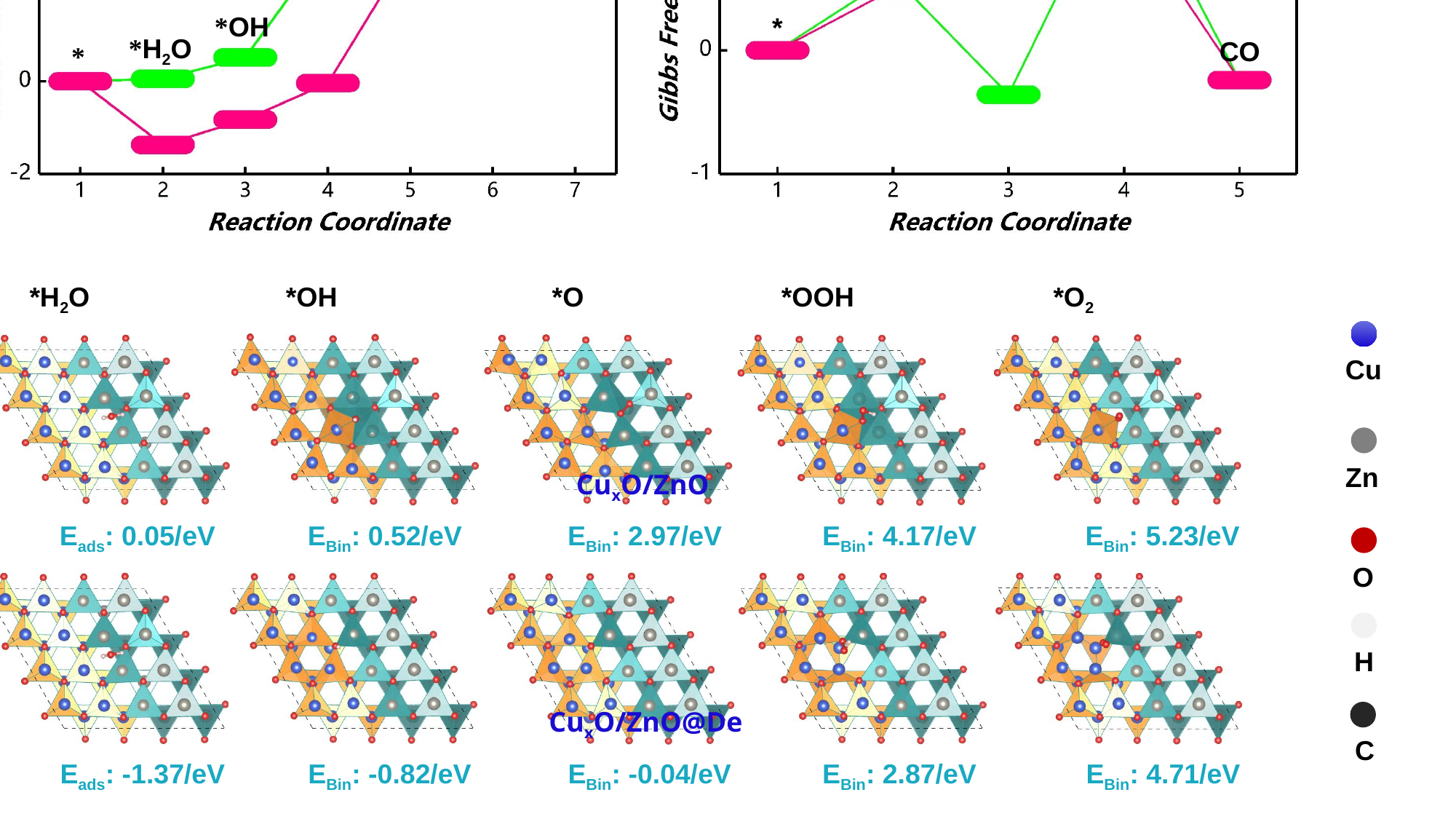

(a)
*O2
O2
*CO
*OOH
*COOH
*O
*CO2
*OH
*
*H2O
CO
*
(b)
*H2O
*OH
*O
*OOH
*O2
Cu
Zn
CuxO/ZnO
Eads: 0.05/eV
EBin: 5.23/eV
EBin: 0.52/eV
EBin: 2.97/eV
EBin: 4.17/eV
O
H
CuxO/ZnO@De
C
Eads: -1.37/eV
EBin: -0.82/eV
EBin: -0.04/eV
EBin: 2.87/eV
EBin: 4.71/eV
(c)
*CO2
*COOH
*CO
*CO2
*COOH
*CO
CuxO/ZnO@De
CuxO/ZnO
Eads: 0.60/eV
EBin: -0.36/eV
Eads: 0.50/eV
EBin: 0.92/eV
EBin: 1.54/eV
EBin: 1.15/eV

## Slide 5
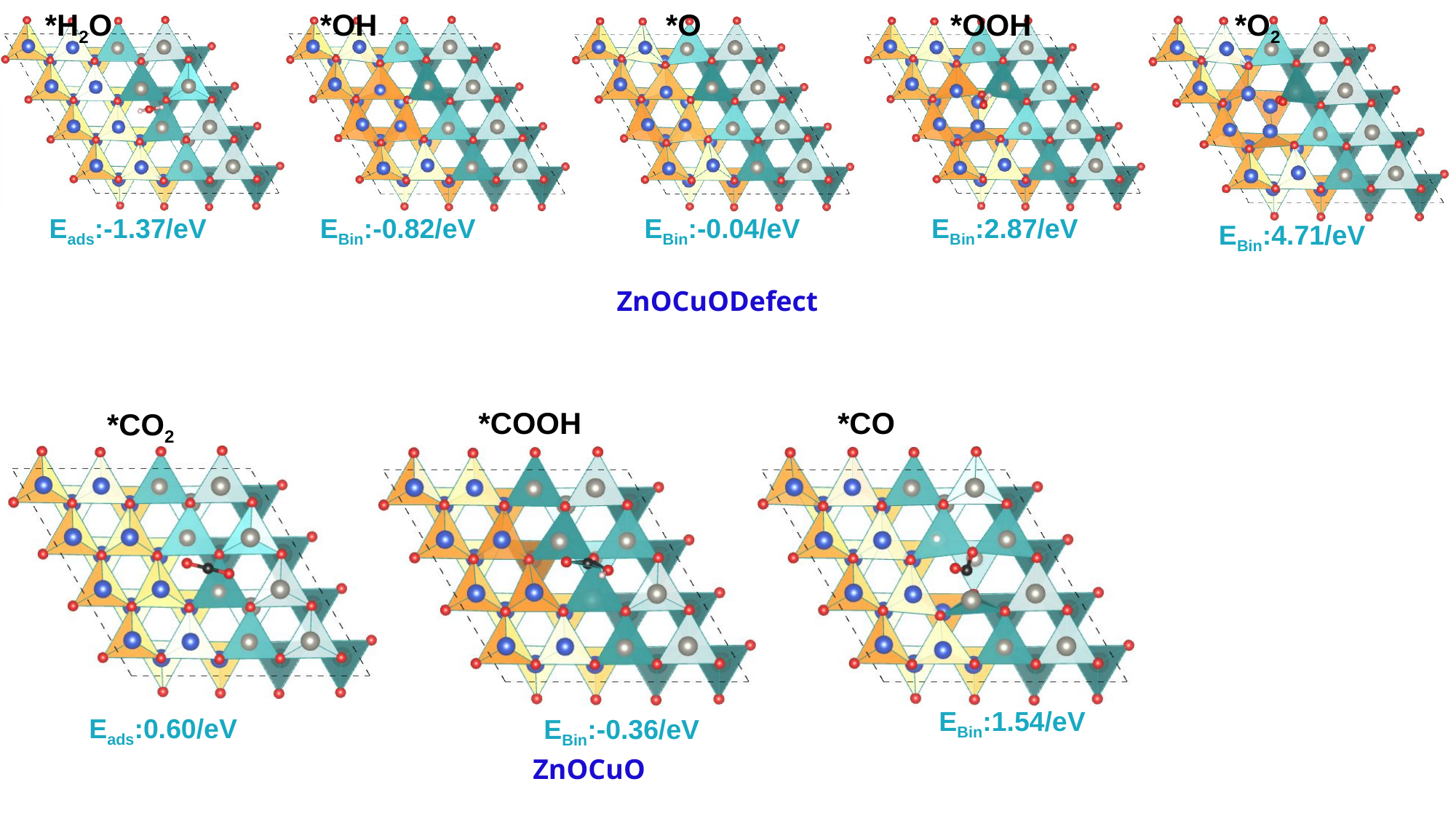

*H2O
*OH
*O
*OOH
*O2
Eads:-1.37/eV
EBin:-0.82/eV
EBin:-0.04/eV
EBin:2.87/eV
EBin:4.71/eV
ZnOCuODefect
*COOH
*CO
*CO2
EBin:1.54/eV
Eads:0.60/eV
EBin:-0.36/eV
ZnOCuO

## Slide 6
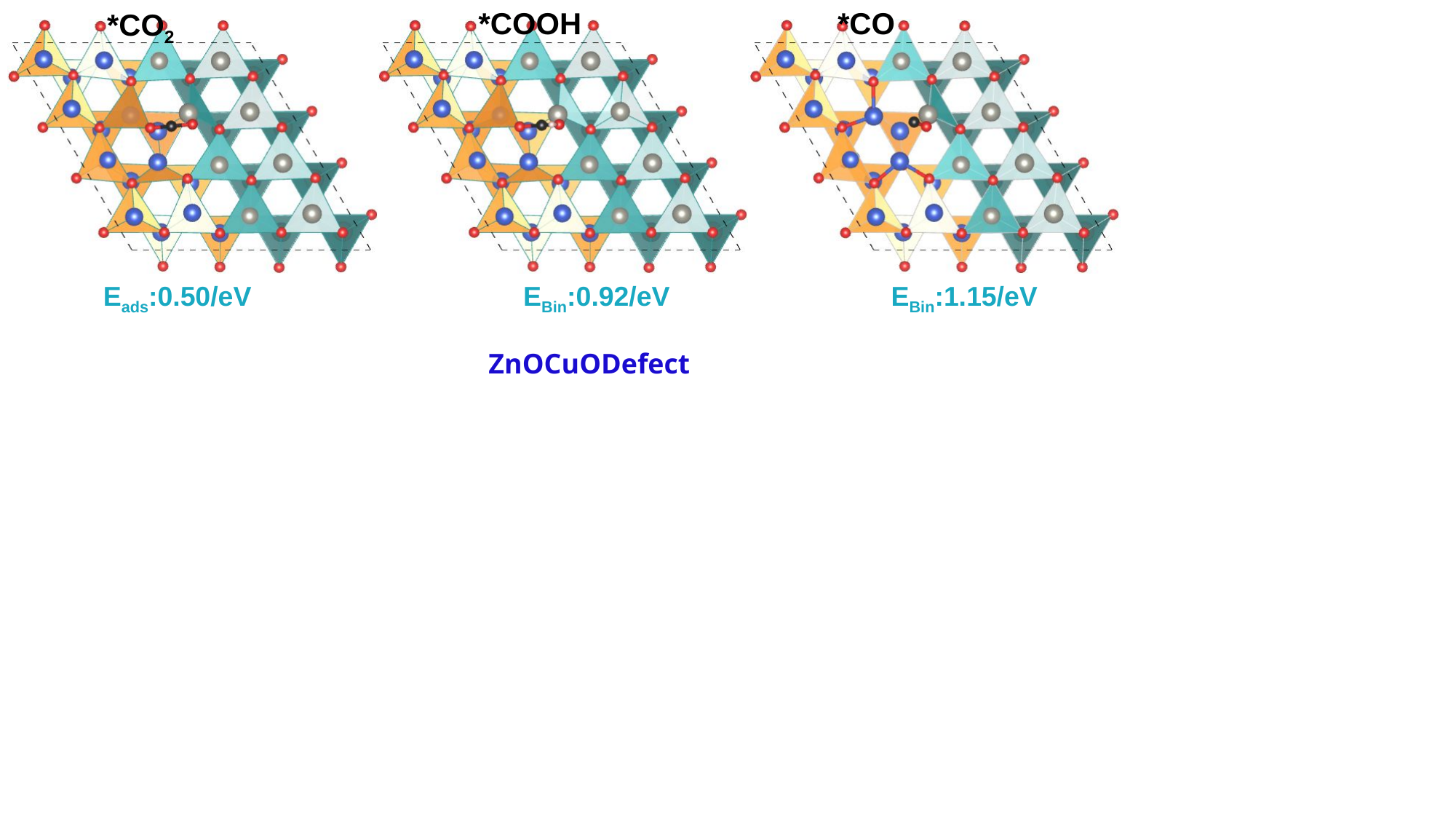

*CO2
*COOH
*CO
Eads:0.50/eV
EBin:0.92/eV
EBin:1.15/eV
ZnOCuODefect

## Slide 7
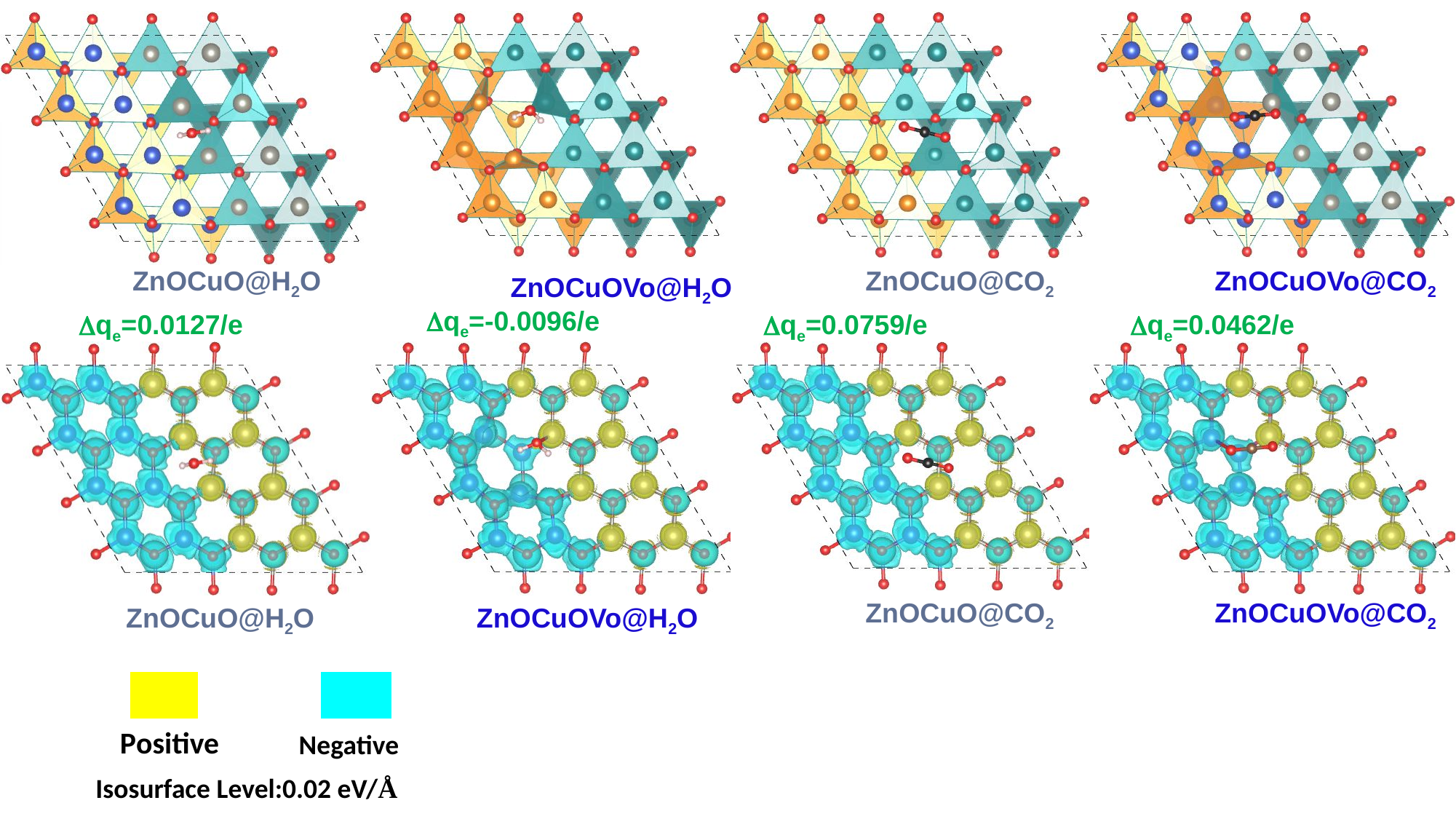

ZnOCuO@H2O
ZnOCuO@CO2
ZnOCuOVo@CO2
ZnOCuOVo@H2O
Δqe=-0.0096/e
Δqe=0.0127/e
Δqe=0.0759/e
Δqe=0.0462/e
ZnOCuO@CO2
ZnOCuOVo@CO2
ZnOCuO@H2O
ZnOCuOVo@H2O
Positive
Negative
Isosurface Level:0.02 eV/Å

## Slide 8
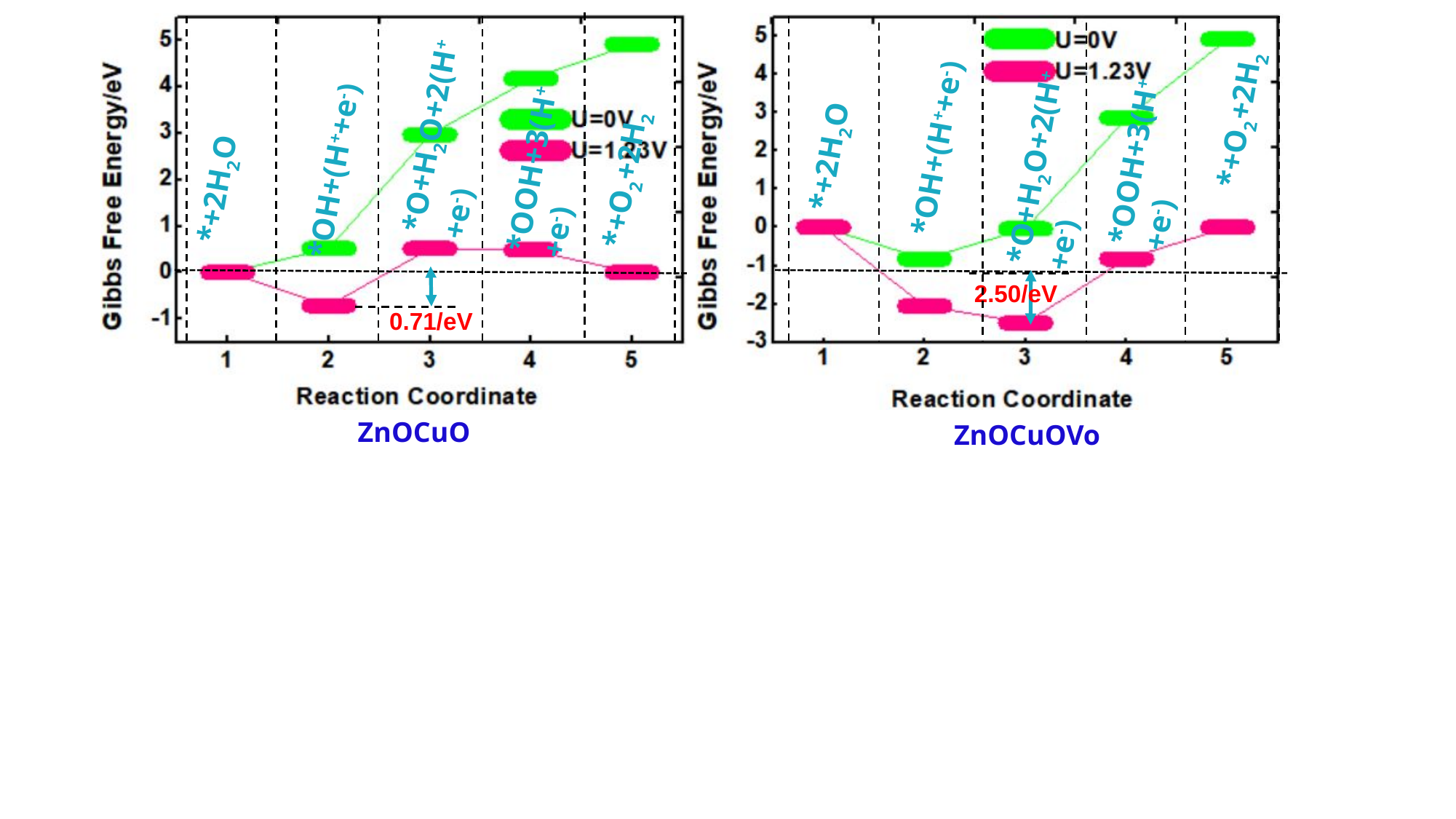

*+2H2O
*+O2+2H2
*O+H2O+2(H++e-)
*OH+(H++e-)
*OOH+3(H++e-)
*+2H2O
*OOH+3(H++e-)
*O+H2O+2(H++e-)
*OH+(H++e-)
*+O2+2H2
2.50/eV
0.71/eV
ZnOCuO
ZnOCuOVo

## Slide 9
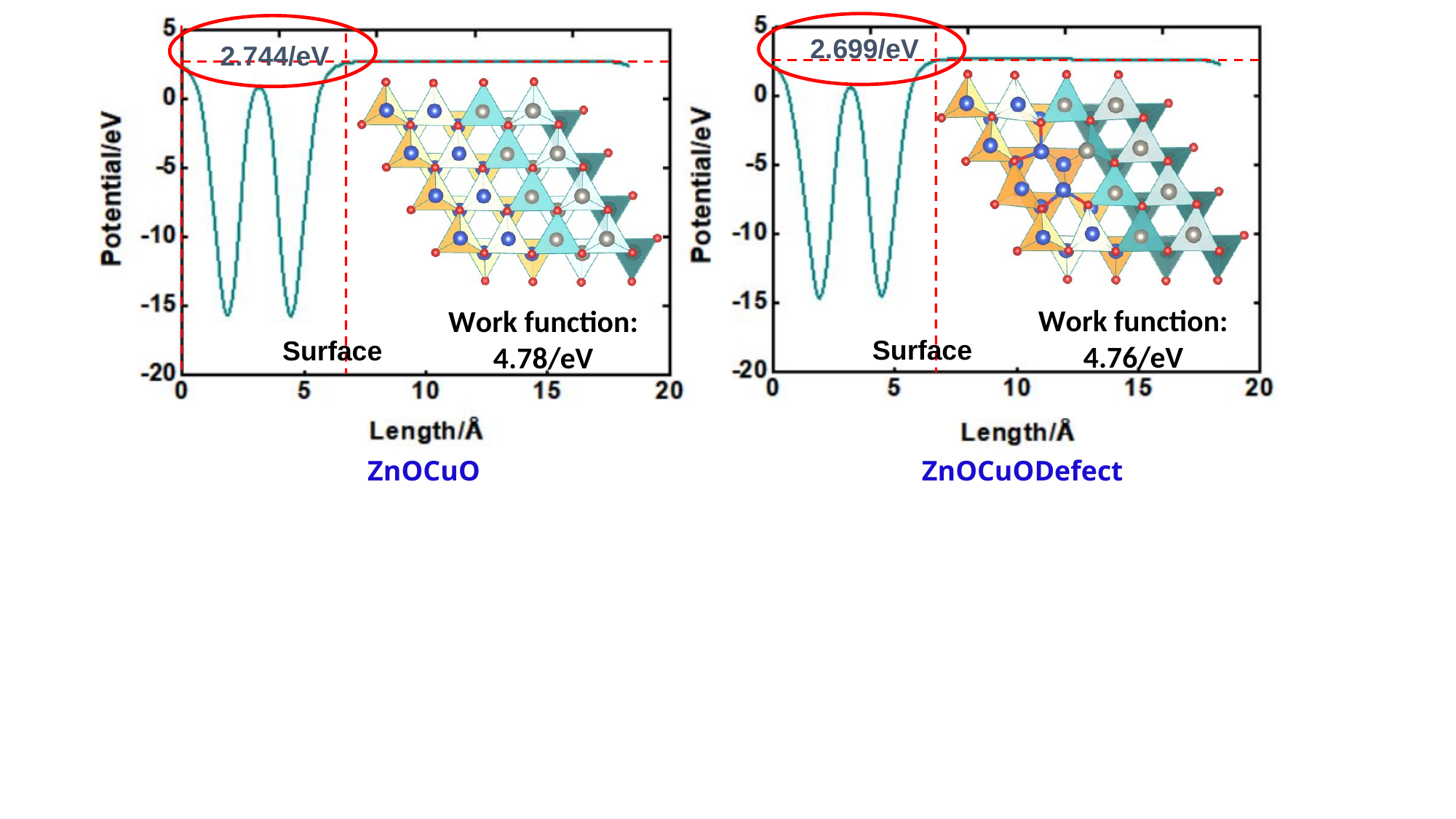

2.699/eV
2.744/eV
Work function:
4.76/eV
Work function:
4.78/eV
Surface
Surface
ZnOCuO
ZnOCuODefect

## Slide 10
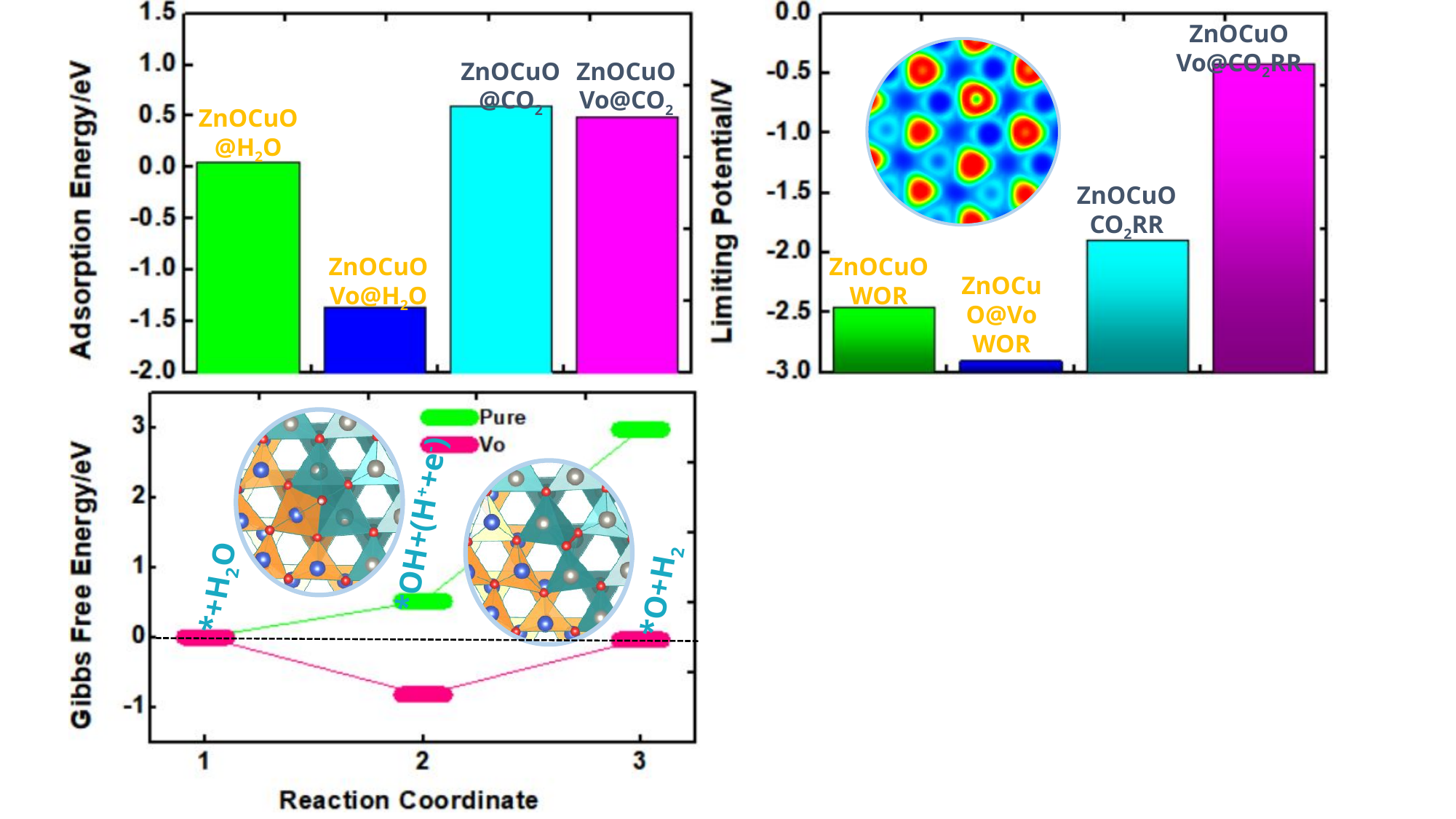

ZnOCuO
Vo@CO2RR
ZnOCuO
@CO2
ZnOCuO
Vo@CO2
ZnOCuO
@H2O
ZnOCuO
CO2RR
ZnOCuO
Vo@H2O
ZnOCuO
WOR
ZnOCuO@Vo
WOR
*OH+(H++e-)
*O+H2
*+H2O

## Slide 11
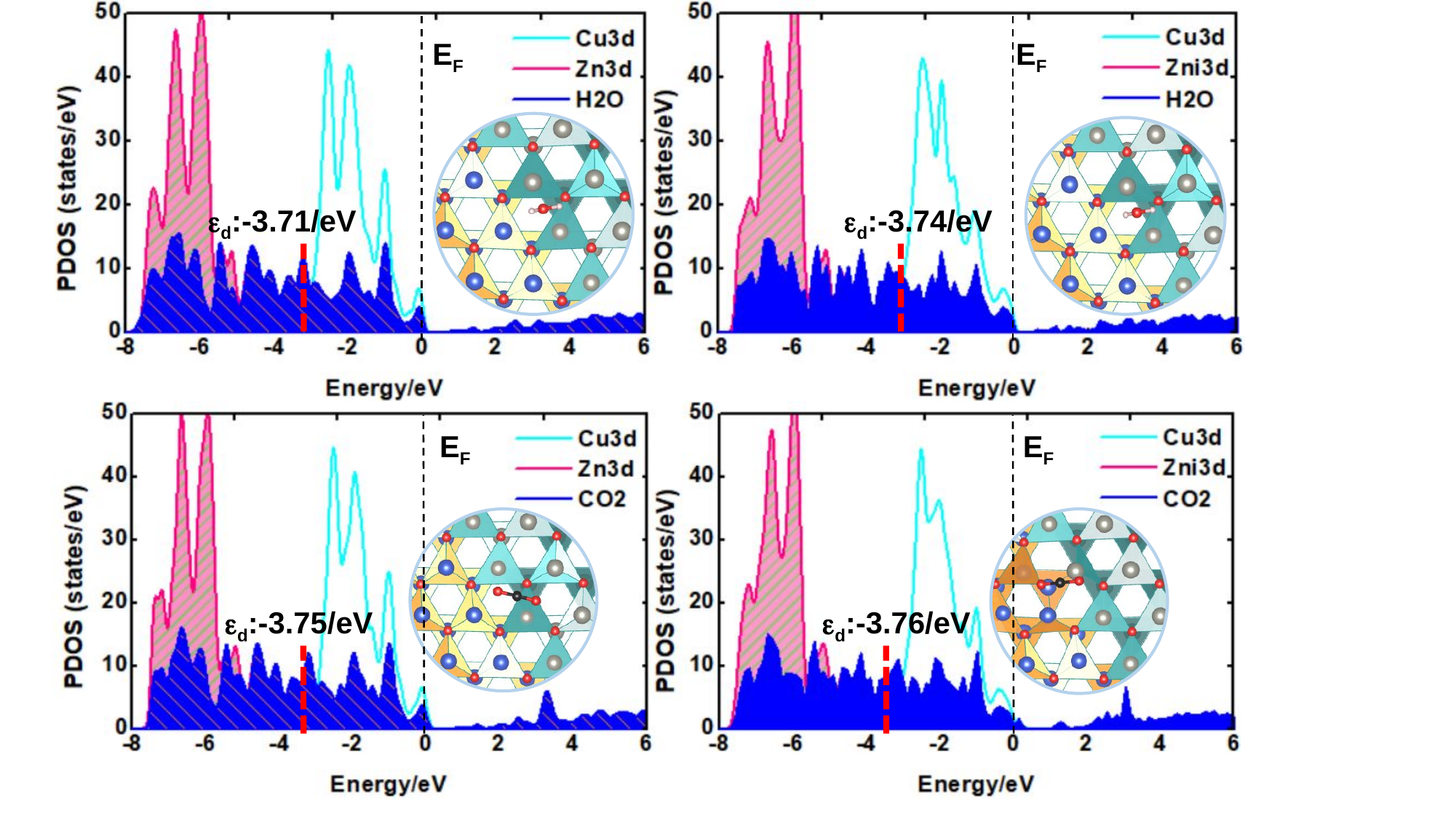

EF
EF
εd:-3.71/eV
εd:-3.74/eV
EF
EF
εd:-3.75/eV
εd:-3.76/eV
